# Supplementary material for: Enhancing authenticity, diagnosticity and equivalence (AD-Equiv) in multicentre OSCE exams in health professionals education: protocol for a complex intervention study
Source: BMJ Open. 2022 Dec 7;12(12):e064387. doi: 10.1136/bmjopen-2022-064387 (PMC9730346; doi:10.1136/bmjopen-2022-064387)
Supplement: Author's manuscript [file bmjopen-2022-064387.draft_revisions.pdf]

BMJ Open is committed to open peer review. As part of this commitment we make the peer review history of every article we publish publicly available.

When an article is published we post the peer reviewers' comments and the authors' responses online. We also post the versions of the paper that were used during peer review. These are the versions that the peer review comments apply to.

The versions of the paper that follow are the versions that were submitted during the peer review process. They are not the versions of record or the final published versions. They should not be cited or distributed as the published version of this manuscript.

BMJ Open is an open access journal and the full, final, typeset and author-corrected version of record of the manuscript is available on our site with no access controls, subscription charges or pay-per-view fees (http://bmjopen.bmj.com).

If you have any questions on BMJ Open's open peer review process please email info.bmjopen@bmj.com

BMJ Open

Enhancing Authenticity, Diagnosticity and Equivalence (AD-Equiv) in multi-centre OSCE exams in Health Professionals Education. Protocol for a Complex Intervention Study

Journal:	*BMJ Open*
Manuscript ID	bmjopen-2022-064387
Article Type:	Protocol
Date Submitted by the Author:	04-May-2022

Complete List of Authors:	Yeates, Peter; Keele University, School of Medicine<br>Maluf, Adriano; Keele University, School of Medicine<br>Kinston, Ruth; Keele University, School of Medicine<br>Cope, Natalie; Keele University, School of Medicine<br>McCray, Gareth; Keele University, School of Primary, Community and Social Care<br>Cullen, Kathy; Queen's University Belfast, School of Medicine, Dentistry and Biomedical Sciences<br>O'Neill, Vikki; Queen's University Belfast, School of Medicine, Dentistry and Biomedical Sciences<br>Cole, Aidan; Queen's University Belfast, School of Medicine, Dentistry and Biomedical Sciences<br>Goodfellow, Rhian; Cardiff University, School of Medicine<br>Vallender, Rebecca; Cardiff University<br>Chung, Ching-Wa; University of Aberdeen, School of Medicine, Medical Sciences and Nutrition<br>McKinley, Robert; Keele University, School of Medicine<br>Fuller, Richard; The Christie NHS Foundation Trust<br>Wong, Geoff; UCL, Research Department of Open Learning

Keywords:	MEDICAL EDUCATION & TRAINING, QUALITATIVE RESEARCH, STATISTICS & RESEARCH METHODS

SCHOLARONE™
Manuscripts

Enhancing Authenticity, Diagnosticity and Equivalence (AD-Equiv) in multi-centre OSCE exams in Health Professionals Education. Protocol for a Complex Intervention Study

Peter Yeates (p.yeates@keele.ac.uk), Adriano Maluf (a.maluf@keele.ac.uk), Ruth Kinston (r.kinston@keele.ac.uk), Natalie Cope (n.a.cope@keele.ac.uk), Gareth McCray (g.mccray@keele.ac.uk), Kathy Cullen (k.cullen@qub.ac.uk), Vikki O'Neill (vikki.oneill@qub.ac.uk), Aidan Cole (a.cole@qub.ac.uk), Rhian Goodfellow (goodfellow@cardiff.ac.uk), Rebecca Vallenderr (vallenderr1@cardiff.ac.uk), Ching-Wa Chung (wa.chung@abdn.ac.uk), Robert McKinley (r.k.mckinley@keele.ac.uk), Richard Fuller (richardfuller@nhs.net), Geoff Wong (geoffrey.wong@phc.ox.ac.uk).

Correspondence to Dr Peter Yeates (School of Medicine, Keele University): p.yeates@keele.ac.uk

Abstract:

Introduction: Objective structured clinical exams (OSCEs) are a cornerstone of assessing healthcare trainees' competence, but have been criticised for a/ lacking authenticity, b/ variability in examiners' judgements which can challenge assessment equivalence and c/ for limited diagnosticity of trainees' focal strengths and weaknesses. This study investigates whether a/ sharing integrated-task OSCE stations across institutions can increase perceived authenticity, whilst; b/ enhancing assessment equivalence by enabling comparison of the standard of examiners' judgements between institutions using a novel methodology (VESCA); and c/ exploring the potential to develop more diagnostic signals from data on students' performances.

Methods and Analysis: This study uses a complex intervention design, developing, implementing and sharing an integrated-task (research) OSCE across four UK medical schools. It employs "Video-based Score Comparison and Adjustment" (VESCA) to compare examiner scoring differences between groups of examiners and different sites, whilst studying how, why and for whom the shared OSCE and VESCA operates across participating schools. Quantitative analyses comprise Many Facet Rasch Modelling to compare the influence of different examiner groups and sites on students' scores,

For peer review only - http://bmjopen.bmj.com/site/about/guidelines.xhtml

whilst the operation of the two interventions (shared integrated task OSCEs; VESCA) will be studied through the theory-driven method of Realist Evaluation.

Ethics: All participation will be voluntary, upholding principles of informed consent, the right to withdraw, confidentiality and data security. The study has received ethical approval from Keele University Research Ethics Committee (Ref: MH-210209)

Dissemination: findings will be academically published and will contribute to good practice guidance on 1/ the use of VESCA and 2/ sharing and use of integrated-task OSCE stations.

For peer review only

Strengths and Limitations:

- The study concurrently addresses three important current considerations relating to the practice of OSCEs in health professionals' education: authenticity, equivalence and performance diagnosticity.
- The study uses a complex intervention design to explain how two separate interventions operate when jointly shared across medical schools to address authenticity and equivalence: a/ integrated-task OSCE stations and b/ video-based examiner score comparison and adjustment (VESCA).
- The study will further examine resulting score data to determine whether diagnostic signals can be determined on different domains of student performance.
- Whilst the research context in which these interventions operate could differ from use in routine practice, this study will provide sufficient insight to enable further evaluation of the interventions in routine practice.

For peer review only - http://bmjopen.bmj.com/site/about/guidelines.xhtml

Introduction

Dependable assessment of the performance and skills of graduating health professionals (doctors, nurses, physiotherapists, pharmacists etc) remains critical to ensuring fairness for students(1) and patient safety(2,3). OSCEs generally involve students rotating around a carousel of timed, simulated clinical tasks being observed on each task by different, trained, examiners who score performances using specified criteria (4). Over recent decades, Objective Structured Clinical Exams (OSCEs) have become one of the pre-eminent methods of assessing clinical skills performance(5) due to their ability to ensure students are directly observed (6) under equivalent conditions (7) according to an appropriate assessment blueprint(8) whilst avoiding some of the limitations of workplace assessments such as case selection, impression management(9), or prior performance information(10).

Despite these benefits, OSCEs have been criticised for:

- Lacking authenticity
- Examiner variability, which can challenge equivalence
- Limited ability to ensure that students are competent in all skills domains

The authenticity of OSCEs has been criticised due to their simulated context and task fragmentation (11,12), which in turn could challenge the applicability of their outcomes to clinical practice(13). In response, several institutions have explored use of OSCE stations which combine multiple tasks (14) – termed “integrated task OSCEs” or greater levels of simulation fidelity (15) to more closely mimic real practice. Whilst these appear to offer a promising development, it is unclear how the added complexity of these tasks influences examiners’ judgements and therefore OSCE standardisation.

Furthermore, examiner variability in OSCEs continues to be significant(16). Owing to student numbers, OSCE exams are often run across several ostensibly identical parallel versions of the same

For peer review only - http://bmjopen.bmj.com/site/about/guidelines.xhtml

exam or distributed across geographical locations, with different examiners in each parallel version. Several studies suggest potentially important differences between the different cohorts of examiners in each parallel version of the exam within single institutions(17) or in large scale distributed exams (18,19). Whilst these variations could compromise the fairness or safety of the resulting assessment decisions, they are rarely studied due to difficulties in directly measuring the influence of unlinked groups of examiners in different parallel versions of the exam. Consequently, little is known about how regional variations in examiners' judgements might challenge the equivalence of OSCEs (20) which could produce different outcomes for students in OSCE exams.

Two pre-requisites are necessary to determine equivalence within a distributed OSCE: firstly, common (or shared) OSCE content is needed, in order for examiners' judgements to be comparable, and secondly, a method is needed to compare examiners' scoring when they are distributed across different locations. In the UK, medical schools set their own OSCE exams, resulting in variation in content and format between Schools. Consequently, sharing OSCE content between schools, whilst necessary, will involve change from usual practice which could further influence examiner variability or produce unintended consequences.

Recently, Yeates et al (21–23) have iteratively developed a method to compare examiners' scoring within distributed OSCEs, called Video-based Examiner Score Comparison and Adjustment (VESCA). This produces linking of otherwise unlinked groups of examiners (termed "examiner-cohorts"(17) by 1/ videoing a small subset of students on each station of the OSCE; 2/ asking examiners from all examiner-cohorts to score the same station-specific comparator videos; and 3/ using the resulting score linkage to compare and equate for differences in examiner-cohorts. Their findings suggest that despite following accepted procedures for OSCE conduct, significant differences may persist between groups of examiners which could affect the pass/fail classification of a significant minority of students. Follow-up work has enhanced the technique's feasibility (23), and shown that it is adequately robust to several potential confounding influences (24) and variations in implementation

For peer review only - http://bmjopen.bmj.com/site/about/guidelines.xhtml

(25). Whilst these findings suggest that examiner-cohort effects are important and support the validity of VESCA for their measurement, VESCA has not yet been used across institutions, so both the likely magnitude of effects which may arise, and the practical implications of applying the method across institutions are unknown.

Finally, recent inquiry has focused on ensuring that trainees are competent across all relevant domains of performance(26) rather than simply demonstrating a sufficient total score, as is often the case in OSCEs (27). This had led to scrutiny of the ability of OSCEs to prevent compensation between domains (28) and whether OSCEs could provide greater diagnosticity of students' areas of focal weakness. Whilst non-compensatory domain-based scoring has been trialled in other arenas (29), little is known about the psychometric properties of such domain scores or whether they can provide independent reliable scores for the constructs they represent. As the utility of VESCA would be greatly enhanced by providing domain level information which has been adjusted for the examiner-cohort effects, it is desirable to study the potential for these data to provide that information.

Aims and Objectives:

This project has a series of aims, objectives and research questions that set out to address the criticisms described above about OSCE examinations. These are:

Criticism 1: Lack of authenticity.

- Objective 1: to increase perceived authenticity of an OSCE through use of integrated-task OSCE stations.

Criticism 2: Examiner variability and challenges to equivalence.

- Objective 2: to share integrated-task OSCE stations across multiple institutions and understand the implications which arise.

For peer review only - http://bmjopen.bmj.com/site/about/guidelines.xhtml

- Objective 3: to use the VESCA methodology for the first time, within the context of an integrated-task OSCE which is shared across multiple institutions, to
    a. compare and equate for differences between examiner-cohorts in different institutions and
    b. understand the implications which arise from using VESCA across institutions.

Criticism 3: Limited diagnosticity of OSCEs across different domains of performance.

- Objective 4: to determine whether different domains of performance can be reliably distinguished from score data within a shared integrated-task OSCE.

Research Questions

Objectives 1 and 2 will be addressed jointly through research question 1:

When integrated-task stations are used and shared within an OSCE, how, when, why and to what extent do examiners, students and simulated patients use and interact with them and how does this influence their perception of the authenticity of the OSCE scenarios?

Objective 3a will be addressed by the following research questions 2-5:

2. How does the standard of examiners' judgements compare between examiner-cohorts?
3. How does the standard of examiners' judgements compare between institutions?
4. What are the relative magnitudes of inter versus intra institutional variation?
5. How much influence does adjusting for examiner-cohort effects have on students':
    a. Overall Scores
    b. Categorisation (fail / pass / excellence)
    c. Rank position

Objective 3b will be addressed through research question 6:

For peer review only - http://bmjopen.bmj.com/site/about/guidelines.xhtml

When VESCA is used to compare and equate for differences between examiner-cohorts in different institutions within the context of a shared integrated-task OSCE, how, when, why and to what extent do examiners, students and simulated patients use and interact with VESCA?

Objective 4 will be addressed through research questions 7-8:

7. How reliably can different domains of assessment be discriminated in unadjusted data?
8. Do students show differing patterns of performance across different domains of the assessment in unadjusted data?

Methods

Methodological Overview:

The study will use a complex intervention design(30) to implement Video-based Examiner Score Comparison and Adjustment (VESCA) in the context of a multi-centre authentic-task OSCE. Research approaches will comprise psychometric analysis of assessment data(31) and Realist evaluation(32), collecting data through mixed methods. A schematic overview of the data collection and analysis is provided in Figure 1.

Population, Sampling and Recruitment:

The study population will comprise participants of late years (penultimate and final year) undergraduate medical student clinical exams within the United Kingdom.

This population will be sampled by recruiting four medical schools to participate as centres in the study, with sampling from all relevant examiners, students, simulated patients. As no prior work has formally compared OSCE examination standards across UK medical schools, the study will aim to sample across different characteristics which might plausibly influence scoring: geographic divergence; Russell group and non-Russell group Universities; and new and more established medical schools.

For peer review only - http://bmjopen.bmj.com/site/about/guidelines.xhtml

Recruitment will be performed locally by each participating institution using both in-person and electronic advertisements. Each participating institute will have recruitment targets for students (n=24), examiners (n=12), and simulated patients (n=12). This sample size is pragmatic based on the resource implications for individual institutions of running a research OSCE. Whilst no formal method exists to power comparisons, or any agreed minimally important difference for differences between groups of OSCE examiners, subset analysis of data from Yeates et al 2021(23) suggests this sample size is likely to provide a standard error in the region of 0.03 logits, enabling statistically significant detection of a difference between examiner cohorts of 5% of the assessment scale.

OSCE Design:

The OSCE comprises six 13.5 minutes tasks (stations), with additional time to rotate between stations of between 1.5 – 4 minutes, depending on each school's usual practice. Station content (simulated patient scenarios / instructions / stimulus materials / scoring rubrics) will be developed by the research team to reflect plausible simulated scenarios from Foundation year 1 doctors routine work and integrate multiple related processes which would be required for whole-task completion. The same stations will be used in all 4 study sites, whilst allowing minor adaptation for local contexts (for example by providing local antibiotic guidelines or dosage calculators).

Individual students will rotate around all 6 stations, and be observed by a different, single examiner in each station during a 90 minute "cycle" of the exam. Each site will host two parallel circuits of the OSCE (identical OSCE stations, run with different examiners). Twelve students will be examined in each parallel circuit (i.e. two cycles of 6 students), enabling 24 students to be tested at each site.

Examiners will be provided with station material (clinical scenario, simulated patient script, marking criteria) prior to the OSCE. Additionally, examiners will be provided with a web-link to a training video which will orientate them to the scoring format.

For peer review only

For peer review only - http://bmjopen.bmj.com/site/about/guidelines.xhtml

Examiners at all sites will score students' performances on the GeCoS rating system(33) with appropriate domains selected for each station to reflect the station's content. Scoring will use tablet or paper-based marking based on available resources at each site.

The OSCE will be conducted first at the lead site (Keele) to enable video production for VESCA procedures; timing in other institutions will vary within eight months to fit with local curricular demands. Local site teams will operationalise their station content based on the constraints of their local resources and equipment. Timing of stations will use local timing facilities but will adhere to standard timing intervals.

Intervention

VESCA will be employed using the methods developed by Yeates et al (21–23).

Video filming: Performances of all students in all 6 stations, from the first cycle, on a selected circuit, will be filmed at the lead site (Keele) using methods established by Yeates et al (22). Filming will use two unobtrusive wall-mounted closed-circuit TV cameras in every room (ReoLink 432, 1080 HD resolution). Camera position, angle and zoom will be selected to optimise capture of the performance. Sound will be recorded using a stereo condensing boundary microphones (Audio-Technica Pro 44). The first three videos from each station which are technically adequate (unobstructed pictures with adequate sound) will be selected and processed for further use, resulting in three comparison videos for each of the six stations in the OSCE.

Video scoring: Examiners will be asked to score the three selected videos selected for the station they examine. All examiners who examine a given station will score the same videos. Consistent with Yeates et al(23), scoring will be performed on-line via a secure survey system including the following elements: on-line consent; station-specific examiner information; sequential presentation of the 3 comparison videos for the station. Examiners will have to score each video and provide brief

For peer review only - http://bmjopen.bmj.com/site/about/guidelines.xhtml

For peer review only

feedback before progressing to the next. As per Yeates et al (22), examiners will have 4 weeks after the OSCE to complete video scoring.

Data Collection:

Student scores (live and video performances) from each site will be collated and labelled with unique identifiers indicating 1/ student, 2/ site, 3/ circuit, 4/ station, 5/examiner, 6/ examiner-cohort and 7/ video or live performance. These data will be used to address all psychometric research questions.

To address RQs_1&6, researchers will develop an initial programme theory (IPT)(34) to orientate and focus subsequent data collection and analysis. To develop the initial programme theory, researchers will consider prior research on VESCA, published experiences of international multi-institutional OSCE collaborations, formal theories which concern institutional adoption of innovations, and the views of a range of experience assessment professionals.

Data will be collected iteratively, interspersed by analysis(35), through a mixture of observation, individual interviews (36) and (where feasible) focus groups, supplemented by available process data. This, along with score data, will be triangulated across modalities to support validity.

Interviews will sample individuals from all relevant stakeholder groups at each site, focused on individuals who have participated in the research OSCE. Whilst sampling requirements will be data driven, indicative numbers of each group from each site are students (n=4), examiners (n=4), simulated patients(n=3), and OSCE administrators(n=1-2). All individuals participating in the OSCE will be invited to be interviewed. If offers of participation exceed sampling needs, then participants will be selected to maximise sample representativeness.

Recruitment will be performed by email. Participation will be voluntary. Participants will receive study information and asked to record their consent through an on-line consent form. Interviews will be conducted by members of the research team (PI, or research assistants), and are expected to last

For peer review only - http://bmjopen.bmj.com/site/about/guidelines.xhtml

45-60 mins. Interviews will be conducted in-person in a private place or via Microsoft Teams. Interviews will be audio recorded and professionally transcribed. Interviews will be guided by a topic guide which will draw from the IPT and evolving theory and will be illustrated by practice-based examples where needed. The interview approach will be adapted to glean, refine and then consolidate emerging theory (37).

Two researchers will observe the “on-the-day” conduct of the OSCE in each participating medical school, using Realist ethnographic observation methods (38). Observations will include the preparation for the OSCE, station layout, equipment set-up, timing and scoring methods; OSCEs conduction, including student flow around the circuits and observation of students examiners and simulated patients behaviour and interactions during and between station performances; students and examiners interaction with filming; and participants’ responses to both the OSCE and VESCA in breaks or after the OSCE is complete. Researchers’ observations will be recorded through fieldnotes.

Public Involvement

Patients and members of the public have been involved throughout the VESCA programme of research which has led to this study. This has included establishing the priority of the research, reviewing plain English summaries, contributing to the design of the research, reviewing progress contributing to elements of the analysis and interpreting findings and discussing future directions. Members of the public are expected to contribute to dissemination activities.

Analysis:

Realist Analyses (used for data relating to RQs_1&6).

Similar analysis methods will be used for both questions. Audio recordings of interviews and focus groups will be professionally transcribed. Observation field notes, where available, will be incorporated into the dataset as will summaries of score data, participation rates and engagement metrics from on-line video scoring by examiners and video access metrics from the on-line feedback

For peer review only - http://bmjopen.bmj.com/site/about/guidelines.xhtml

portal for students. Analysis will use the stages described by Papoutsi et al (45). This begins by reading or considering each piece of data line-by-line to judge its relevance to the initial programme theory. Decisions will be made about the trustworthiness of relevant data. Next, researchers will allocate initial conceptual labels. Conceptual labels will be derived both deductively from the initial programme theory and inductively based on researchers' interpretation of emergent issues. Researchers will consider whether each labelled concept can be interpreted to represent a context (C), a mechanism (M) or an outcome (O) and will look for data which provides information on the relatedness of Cs, Ms, and Os with the intent to develop Context-Mechanism-Outcome-Configurations (CMOC). Drawing on relevant data, researchers will interpret how each CMOC relates to the programme theory and iteratively revise the programme theory as more and more CMOCs are developed. Interpretation will use the analytic processes of juxtaposition, reconciliation, adjudication and consolidation to explore discrepancies and resolve differences. Interpretation will also use retro-duction, combining both induction based on emergence from the data and deduction from the initial programme theory in order to unearth mechanistic relations within CMOCs and the Programme theory(46,47). Analysis will proceed iteratively, interspersed with new data collection until a coherent and plausible programme theory is reached.

Psychometric analyses (used for RQs_2-5, 7,8)

RQs_2-5 will be addressed using Many Facet Rasch Modelling (MFRM), conducted using FACETs by Winsteps (39). The dependent variable for analyses will be denoted "total score" and will be calculated for each student on each station by combining the scores for each domain. Categorical independent variables will be available for each station score, describing the student (ID number); station (number); examiner (ID number); examiner-cohort (ex-cohort ID); and site (institution ID). These data will be analyses using a four facet Rasch model, with facets of: 1/ student, 2/ station, 3/ examiner-cohort and 4/ site.

For peer review only - http://bmjopen.bmj.com/site/about/guidelines.xhtml

To ensure data are adequate for MFRM analysis, research will assess the dimensionality, ordinality and model-fit of data. Dimensionality will be assessed using principle components analysis (PCA) of model residuals with random parallel analysis using R studio for R(40). Ordinality of the scale will be determined by examining the Rasch-Andrich thresholds supplied in FACETs output data(FACETS v3.82.3 Winsteps, Western Australia). Fit parameters supplied by FACETs will be examined to determine data to model fit, using the criteria advocated by Linacre (41). The analysis plan will be adapted if data are inadequate for MFRM analysis by choosing an appropriate alternative method such as linear mixed modelling.

To address RQ_2, observed (Raw score) average scores and "Fair-Average" scores(42) for examiner-cohorts will be compared, and the difference between observed (Raw score) average and Fair average will be calculated for each examiner-cohort and compared. Observed differences will be transformed into multiples of the standard error to calculate statistical significance.

To address RQ_3 observed (Raw score) average scores and "Fair-Average" scores(42) for each site (institution) will be compared and the difference between their observed (Raw score) average and Fair average will be calculated for each site and compared them.

To address RQ_4, the difference between examiner-cohorts within each institution (i.e. site) will be calculated and compared with the differences between the values for different institutions.

To address RQ_5a, the difference between the raw observed average score and the fair average score will be calculated for each participating student. These will be converted to mean absolute differences (MAD) to remove the direction of score adjustment. Descriptive statistics will be calculated for both the raw score adjustments and MAD adjustments. Similar to prior research (21,23), the effect size of each MAD score adjustment will calculated using Cohen's d (43), using the standard deviation of students' average observed scores as the denominator. The mean Cohen's d and the proportion of students' whose adjustment exceeds d=0.5 will be reported.

For peer review only - http://bmjopen.bmj.com/site/about/guidelines.xhtml

To address RQs_5b&c, category boundaries will be developed using the borderline regression method(44) for each station and pooled to give an average cut score for the test. Two separate values will be interpolated from the x-axis: one to represent a fail/pass boundary and one to represent a pass/excellent boundary. Each students' categorisation for the OSCE relative to these boundaries will be determined based on their observed raw average score and their fair average score and the proportion changing categories (number increasing a grade; number reducing a grade) will be calculated for both thresholds. Students rank position in the OSCE (regardless of institutional rankings) will be calculated based on observed raw average scores and fair average scores and the difference between each student's rank position from each score calculated. This will be expressed as both raw change in rank (positive or negative sign) and MAD change in rank which will be summarised through descriptive statistics.

RQs_7&8 represent exploratory forms of analysis. These analyses will employ individual scores domains within each station as dependent variables. Domains will be grouped based on content into dimensions which represent communication skills, knowledge and reasoning, investigation and management and procedural skills. Exploratory Factor Analysis will be applied to determine the level of support for these dimensions, using Cronbach's alpha to estimate the reliability of scores within each dimension. Student-level dimension scores will be examined to produce descriptive statistics describing dimension level scores and to determine the proportion of students who show greater than 0.5 standard deviation score difference between difference dimensions. Further exploratory analyses will determine whether categorical differences exist for some students across domains (i.e. greater frequency of borderline categories in 1 domain).

For peer review only - http://bmjopen.bmj.com/site/about/guidelines.xhtml

Anticipated Outcomes:

Realist evaluations will produce mature programme theories which describe how different contexts elicit different mechanisms to produce varied outcomes for different stakeholders when a/ an integrated authentic task OSCE is shared between medical schools and b/. VESCA is implemented across multiple medical schools. This will be used to produce guidance on successful implementation of both interventions. Realist Evaluations will be reported using the standards of the RAMESES II reporting standards(36).

Psychometric analyses for RQs_2-5 will describe the extent of overall score variability which arose between examiner-cohorts and institutions in the standard of examiners' judgements, and the impact of adjustment for these on students' scores, categorisation and rank.

Psychometric analyses for RQs_7&8 will describe the dimensionality of domain-score data and varied patterns of strength and weakness in students' performances, with comparison in patterns across schools.

Outputs and Dissemination:

Findings of the research will be disseminated through academic publications, conference presentations and workshops and through engagement meetings with educational institutions who may adopt or implement VESCA or Video-based feedback.

<u>Outputs</u>:

Good practice guidelines for the use of VESCA to enhance OSCE examiner standardisation in distributed exams and for sharing integrated task OSCEs across institutions. Intended audiences: institutions, assessment leads, examiners. Engagement work through the Association for the Study of Medical Education Psychometrics Specialist Interest Group (ASME psychoSIG) to promote this to policy makers.

For peer review only

For peer review only - http://bmjopen.bmj.com/site/about/guidelines.xhtml

Explanatory video, describing the purpose, use and benefits of VESCA for a lay audience. Intended audience: students, examiners, members of the public.

The research is expected to produce academic publications describing the following findings:

1. Paper 1: primary psychometric analyses, comparing the influence of examiner-cohorts and institutions on students' scores, categorisation and rank;
2. Paper 2: secondary psychometric analyses, determining the extent of additional diagnostic information available in domain score data;
3. Paper 3: Realist evaluation, a programme theory of the implications of using VESCA within a shared OSCE.

Ethical Considerations

Recruitment will invite the entire target populations of students and examiners in each school, subject to any local exclusions (i.e. adequate academic progress). Simulated patients will participate as per their usual professional working arrangements. Participants will retain the right to withdraw up until their data are anonymised. Researchers will collect personal data to manage recruitment and to link scores from the OSCE, on-line usage and engagement data for video access or scoring and interview and focus group data. These data will be stored securely and treated as confidential. Access will be limited to those members of the research team who require access for the analyses specified within the research. There are few anticipated risks to participants: if videos, score or interview data pertaining to them were disseminated inadvertently then that could cause embarrassment or distress. This risk is mitigated through the confidentiality and data security measures which will be employed. Students may benefit from taking part in the research through the experience of novel OSCE assessment tasks or availability of video feedback. Examiners may benefit from practice at examining. Ethical approval for the study has been granted by Keele University Research Ethics Committee (Ref: MH-210209)

For peer review only - http://bmjopen.bmj.com/site/about/guidelines.xhtml

Anticipated Timeframe:

Developing collaborations: complete by end May 2021.

Finalising protocol: June 2021

Ethics application: July-Sept 2021

OSCE station development: September - October 2021

Scheduling and recruitment of OSCEs: October 2021 – March 2022

Site 1 OSCE: December – March 2022

Sites 2-4 OSCE: January – July 2022

Examiner video scoring: 4-week interval after each OSCE

Interviews / focus groups / observations: December 2021 – August 2022

Psychometric analysis: July – November 2022

Realist analysis February - November 2022

Dissemination: December 2022 - February 2023.

Authors' contributions:

The study design was developed by PY, RK, NC, GM, KC, VO, AC, RG, RV, CWC, LC, RM and RF. PY wrote the original draft. GW provided expertise in Realist Evaluation methodology. PY, AM and NC are collecting data supported by KC, RV, CWC, RG, RV. All authors will contribute to interpreting analysis. All authors critiqued and provided edits to the protocol manuscript for intellectual content.

For peer review only - http://bmjopen.bmj.com/site/about/guidelines.xhtml

Funding statement:

The study is funded through a National Institute for Health Research (NIHR) Clinician Scientist award held by the Principal Investigator, Peter Yeates (NIHR grant number: N/A). The study constitutes independent research and does not represent the views of the NIHR, the NHS or the department of health and social care.

Competing interests:

None.

References:

1. Valentine N, Durning S, Shanahan EM, Schuwirth L. Fairness in human judgement in assessment: a hermeneutic literature review and conceptual framework. Adv Heal Sci Educ. 2020;26(2):713–38.

2. Eva KW. Cognitive Influences on Complex Performance Assessment: Lessons from the Interplay between Medicine and Psychology. J Appl Res Mem Cogn. 2018;7(2):177–88.

3. Kogan JR, Conforti LN, Iobst WF, Holmboe ES. Reconceptualizing variable rater assessments as both an educational and clinical care problem. Acad Med. 2014 May;89(5):721–7.

4. Newble D. Techniques for measuring clinical competence: objective structured clinical examinations. Med Educ. 2004;38:199–203.

5. Boursicot K, Kemp S, Wilkinson T, Findyartini A, Canning C, Cilliers F, et al. Performance assessment: Consensus statement and recommendations from the 2020 Ottawa Conference.

For peer review only - http://bmjopen.bmj.com/site/about/guidelines.xhtml

For peer review only

Med Teach. 2021 Jan 2;43(1):58–67.

6. Harden RM, Stevenson M, Downie WW, Wilson GM. Medical Education Assessment of Clinical Competence using Objective Structured Examination. Br Med J. 1975;1(February):447–51.

7. Norcini J, Anderson MB, Bollela V, Burch V, Costa MJ, Duvivier R, et al. 2018 Consensus framework for good assessment. Med Teach. 2018;0(0):1–8.

8. Wass V, Vleuten C Van Der, Shatzer J, Jones R. Medical education quartet Assessment of clinical competence. Lancet. 2001;357:945–9.

9. Huffman BM, Hafferty FW, Bhagra A, Leasure EL, Santivasi WL, Sawatsky AP. Resident impression management within feedback conversations: A qualitative study. Med Educ. 2021;55(2):266–74.

10. Murto SH, Shaw T, Touchie C, Pugh D, Cowley L, Wood TJ. Are raters influenced by prior information about a learner ? A review of assimilation and contrast effects in assessment. Adv Heal Sci Educ. 2021;(0123456789).

11. Johnston JL, Kearney GP, Gormley GJ, Reid H. Into the uncanny valley: Simulation versus simulacrum? Med Educ. 2020;54(10):903–7.

12. Gormley GJ, Hodges BD, McNaughton N, Johnston JL. The show must go on? Patients, props and pedagogy in the theatre of the OSCE. Med Educ. 2016;50(12):1237–40.

13. Downing SM. Validity: on meaningful interpretation of assessment data. Med Educ. 2003 Sep;37(9):830–7.

14. Ruesseler M, Weinlich M, Byhahn C, Müller MP, Jünger J, Marzi I, et al. Increased authenticity in practical assessment using emergency case OSCE stations. Adv Heal Sci Educ. 2010;15(1):81–95.

For peer review only - http://bmjopen.bmj.com/site/about/guidelines.xhtml

15. Gormley G, Sterling M, Menary A, McKeown G. Keeping it real! Enhancing realism in standardised patient OSCE stations. Clin Teach. 2012;9(6):382–6.

16. Gingerich A. The Reliability of Rater Variability. J Grad Med Educ. 2020;12(2):159–61.

17. Yeates P, Sebok-Syer SS. Hawks, Doves and Rasch decisions: Understanding the influence of different cycles of an OSCE on students' scores using Many Facet Rasch Modeling. Med Teach. 2017;39(1):92–9.

18. Sebok SS, Roy M, Klinger D a, De Champlain AF. Examiners and content and site: Oh My! A national organization's investigation of score variation in large-scale performance assessments. Adv Health Sci Educ Theory Pract. 2015 Aug 28;20(3):581–94.

19. Floreck LM, De Champlain AF. Assessing Sources of Score Variability in a Multi-Site Medical Performance Assessment: An Application of Hierarchical Linear Modeling. Acad Med. 2001;76(10):S93–5.

20. Norcini J, Anderson B, Bollela V, Burch V, Costa MJ, Duvivier R, et al. Criteria for good assessment: consensus statement and recommendations from the Ottawa 2010 Conference. Med Teach. 2011 Jan;33(3):206–14.

21. Yeates P, Cope N, Hawarden A, Bradshaw H, McCray G, Homer M. Developing a video-based method to compare and adjust examiner effects in fully nested OSCEs. Med Educ. 2019 Mar;53(3):250–63.

22. Yeates P, Moult A, Lefroy J, Walsh-House J, Clews L, McKinley R, et al. Understanding and developing procedures for video-based assessment in medical education. Med Teach. 2020 Nov 1;42(11):1250–60.

23. Yeates P, Moult A, Cope N, McCray G, Xilas E, Lovelock T, et al. Measuring the Effect of Examiner Variability in a Multiple-Circuit Objective Structured Clinical Examination (OSCE). Acad Med. 2021 Mar 2;96(8):1189–96.

For peer review only - http://bmjopen.bmj.com/site/about/guidelines.xhtml

For peer review only

24. Yeates P, Moult A, Cope N, McCray G, Fuller R, McKinley R. Determining influence, interaction and causality of contrast and sequence effects in objective structured clinical exams. Med Educ. 2022;56(3):292–302.

25. Yeates P, McCray G, Moult A, Cope N, Fuller R, McKinley R. Determining the influence of different linking patterns on the stability of students' score adjustments produced using Video-based Examiner Score Comparison and Adjustment (VESCA). BMC Med Educ. 2022;22(1):1–9.

26. Frank JR, Snell LS, Cate O Ten, Holmboe ES, Carraccio C, Swing SR, et al. Competency-based medical education: theory to practice. Med Teach. 2010 Aug 27;32(8):638–45.

27. McKinley DW, Norcini JJ. How to set standards on performance-based examinations: AMEE Guide No. 85. Med Teach. 2014;36(2):97–110.

28. Homer M, Russell J. Conjunctive standards in OSCEs: The why and the how of number of stations passed criteria. Med Teach. 2021;43(4):448–55.

29. Pearce J, Reid K, Chiavaroli N, Hyam D. Incorporating aspects of programmatic assessment into examinations: Aggregating rich information to inform decision-making. Med Teach. 2021 Feb 8;0(0):1–8.

30. Craig P, Dieppe P, Macintyre S, Michie S, Nazareth I, Petticrew M. Developing and evaluating complex interventions : new guidance. 2008.

31. Bond T, Fox C. Applying the Rasch Model Fundamental Measurement in the Human Sciences. 2nd Editio. New York & London: Routledge; 2012.

32. Pawson R, Tilley N. Realistic Evaluation. 1st ed. London: Sage Publications Ltd; 1997.

33. Lefroy J, Gay SP, Gibson S, Williams S, McKinley RK. Development and face validation of an instrument to assess and improve clinical consultation skills. Int J Clin Ski. 2011;5(2):115–125.

For peer review only

34. Pawson R, Manzano-Santaella A. A realist diagnostic workshop. Evaluation. 2012;18(2):176–91.

35. Marchal B, van Belle S, van Olmen J, Hoerée T, Kegels G. Is realist evaluation keeping its promise? A review of published empirical studies in the field of health systems research. Evaluation. 2012;18(2):192–212.

36. Wong G, Westhorp G, Manzano A, Greenhalgh J, Jagosh J, Greenhalgh T. RAMESES II reporting standards for realist evaluations. BMC Med. 2016;14(1):1–18.

37. Manzano A. The craft of interviewing in realist evaluation. Evaluation. 2016;22(3):342–60.

38. Hammersley M. Ethnography and Realism. In: Huberman AM, Miles MB, editors. The Qualitative Researcher's Companion. 2455 Teller Road, Thousand Oaks California 91320 United States of America: SAGE Publications, Inc.; 2002. p. 65–80.

39. Linacre JM. Many-Facet Rasch Measurement. 2nd Edicat. Chicago: MESA Press; 1994.

40. Team Rs. RStudio: Integrated Development for R. Boston, MA: RStudio, Inc.; 2015.

41. Linacre JM. What do Infit and Outfit, Mean-square and Standardized mean? [Internet]. Rasch.Org website. 2002 [cited 2018 Jun 12]. p. 16:2, p878. Available from: https://www.rasch.org/rmt/rmt162f.htm

42. Linacre JM. A User's guide to FACETS Rasch-Model Computer Programs. 2005.

43. Cohen J. Statistical Power Analysis for the Social Sciences. 2nd ed. Lawrence Erlbaum Associates; 1988.

44. Downing SM, Tekian A, Yudkowsky R. Procedures for Establishing Defensible Absolute Passing Scores on Performance Examinations in Health Professions Education Procedures for Establishing Defensible Absolute Passing Scores on Performan. Teach Learn Med. 2006;18(1):50–7.

For peer review only - http://bmjopen.bmj.com/site/about/guidelines.xhtml

45. Papoutsi C, Mattick K, Pearson M, Brennan N, Briscoe S, Wong G. Interventions to improve antimicrobial prescribing of doctors in training (IMPACT): a realist review. Heal Serv Deliv Res. 2018;6(10):1–136.

46. Astbury B, Leeuw FL. Unpacking Black Boxes: Mechanisms and Theory Building in Evaluation. Am J Eval. 2010;31(3):363–81.

47. The RAMESES II Project. Retroduction in realist evaluation. Nihr. 2017;(p 207):1–3.

Figure 1: Schematic of the data collection and analysis processes

Word Count: 3996 words

For peer review only - http://bmjopen.bmj.com/site/about/guidelines.xhtml

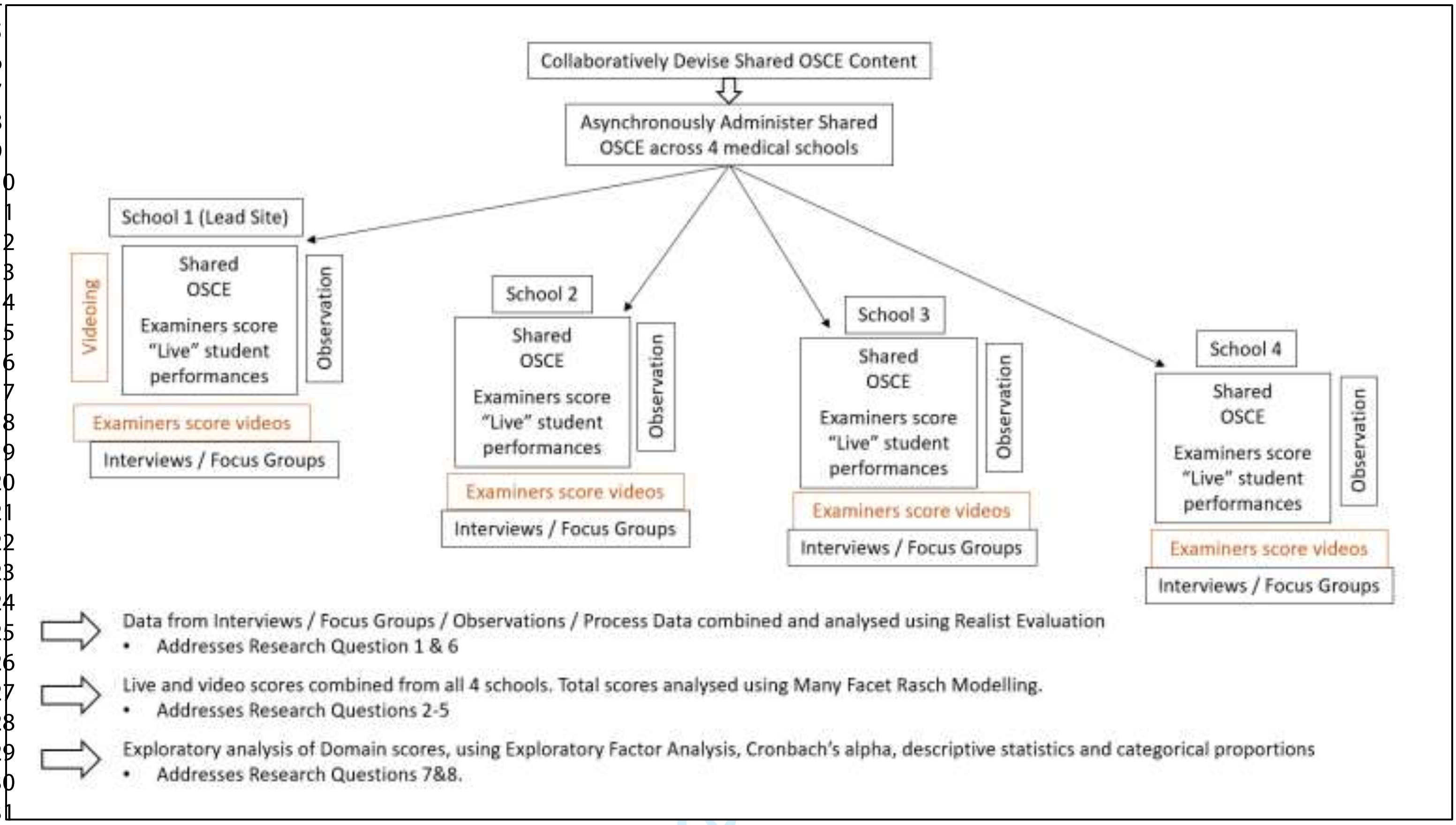
Collaboratively Devise Shared OSCE Content
Asynchronously Administer Shared OSCE across 4 medical schools
School 1 (Lead Site)
Videoing
Shared OSCE
Examiners score "Live" student performances
Observation
Examiners score videos
Interviews / Focus Groups
School 2
Shared OSCE
Examiners score "Live" student performances
Observation
Examiners score videos
Interviews / Focus Groups
School 3
Shared OSCE
Examiners score "Live" student performances
Observation
Examiners score videos
Interviews / Focus Groups
School 4
Shared OSCE
Examiners score "Live" student performances
Observation
Examiners score videos
Interviews / Focus Groups
Data from Interviews / Focus Groups / Observations / Process Data combined and analysed using Realist Evaluation
• Addresses Research Question 1 & 6
Live and video scores combined from all 4 schools. Total scores analysed using Many Facet Rasch Modelling.
• Addresses Research Questions 2-5
Exploratory analysis of Domain scores, using Exploratory Factor Analysis, Cronbach's alpha, descriptive statistics and categorical proportions
• Addresses Research Questions 7&8.

CONSORT 2010 checklist of information to include when reporting a randomised trial*

Section/Topic	Item No	Checklist item	Reported on page No
**Title and abstract**			
	1a	Identification as a randomised trial in the title	1
	1b	Structured summary of trial design, methods, results, and conclusions (for specific guidance see CONSORT for abstracts)	1-2
**Introduction**			
Background and objectives	2a	Scientific background and explanation of rationale	3-5
	2b	Specific objectives or hypotheses	5-7
**Methods**			
Trial design	3a	Description of trial design (such as parallel, factorial) including allocation ratio	7-10
	3b	Important changes to methods after trial commencement (such as eligibility criteria), with reasons	n/a
Participants	4a	Eligibility criteria for participants	10
	4b	Settings and locations where the data were collected	10
Interventions	5	The interventions for each group with sufficient details to allow replication, including how and when they were actually administered	10-11
Outcomes	6a	Completely defined pre-specified primary and secondary outcome measures, including how and when they were assessed	n/a
	6b	Any changes to trial outcomes after the trial commenced, with reasons	n/a
Sample size	7a	How sample size was determined	10-11
	7b	When applicable, explanation of any interim analyses and stopping guidelines	n/a
Randomisation:			
Sequence generation	8a	Method used to generate the random allocation sequence	n/a
	8b	Type of randomisation; details of any restriction (such as blocking and block size)	n/a
Allocation concealment mechanism	9	Mechanism used to implement the random allocation sequence (such as sequentially numbered containers), describing any steps taken to conceal the sequence until interventions were assigned	n/a
Implementation	10	Who generated the random allocation sequence, who enrolled participants, and who assigned participants to interventions	n/a
Blinding	11a	If done, who was blinded after assignment to interventions (for example, participants, care providers, those	n/a

For peer review only - http://bmjopen.bmj.com/site/about/guidelines.xhtml

		assessing outcomes) and how	
	11b	If relevant, description of the similarity of interventions	4
Statistical methods	12a	Statistical methods used to compare groups for primary and secondary outcomes	12-14
	12b	Methods for additional analyses, such as subgroup analyses and adjusted analyses	11-12
**Results**			
Participant flow (a diagram is strongly recommended)	13a	For each group, the numbers of participants who were randomly assigned, received intended treatment, and were analysed for the primary outcome	n/a
	13b	For each group, losses and exclusions after randomisation, together with reasons	n/a
Recruitment	14a	Dates defining the periods of recruitment and follow-up	17
	14b	Why the trial ended or was stopped	n/a
Baseline data	15	A table showing baseline demographic and clinical characteristics for each group	n/a
Numbers analysed	16	For each group, number of participants (denominator) included in each analysis and whether the analysis was by original assigned groups	n/a
Outcomes and estimation	17a	For each primary and secondary outcome, results for each group, and the estimated effect size and its precision (such as 95% confidence interval)	n/a
	17b	For binary outcomes, presentation of both absolute and relative effect sizes is recommended	n/a
Ancillary analyses	18	Results of any other analyses performed, including subgroup analyses and adjusted analyses, distinguishing pre-specified from exploratory	15-16
Harms	19	All important harms or unintended effects in each group (for specific guidance see CONSORT for harms)	16
**Discussion**			
Limitations	20	Trial limitations, addressing sources of potential bias, imprecision, and, if relevant, multiplicity of analyses	n/a
Generalisability	21	Generalisability (external validity, applicability) of the trial findings	15
Interpretation	22	Interpretation consistent with results, balancing benefits and harms, and considering other relevant evidence	11-14
**Other information**			
Registration	23	Registration number and name of trial registry	n/a
Protocol	24	Where the full trial protocol can be accessed, if available	n/a
Funding	25	Sources of funding and other support (such as supply of drugs), role of funders	22

*We strongly recommend reading this statement in conjunction with the CONSORT 2010 Explanation and Elaboration for important clarifications on all the items. If relevant, we also recommend reading CONSORT extensions for cluster randomised trials, non-inferiority and equivalence trials, non-pharmacological treatments, herbal interventions, and pragmatic trials. Additional extensions are forthcoming: for those and for up to date references relevant to this checklist, see www.consort-statement.org.11-114

For peer review only - http://bmjopen.bmj.com/site/about/guidelines.xhtml

BMJ Open

Enhancing Authenticity, Diagnosticity and Equivalence (AD-Equiv) in multi-centre OSCE exams in Health Professionals Education. Protocol for a Complex Intervention Study

Journal:	*BMJ Open*
Manuscript ID	bmjopen-2022-064387.R1
Article Type:	Protocol
Date Submitted by the Author:	12-Sep-2022

Complete List of Authors:	Yeates, Peter; Keele University, School of Medicine<br>Maluf, Adriano; Keele University, School of Medicine<br>Kinston, Ruth; Keele University, School of Medicine<br>Cope, Natalie; Keele University, School of Medicine<br>McCray, Gareth; Keele University, School of Primary, Community and Social Care<br>Cullen, Kathy; Queen's University Belfast, School of Medicine, Dentistry and Biomedical Sciences<br>O'Neill, Vikki; Queen's University Belfast, School of Medicine, Dentistry and Biomedical Sciences<br>Cole, Aidan; Queen's University Belfast, School of Medicine, Dentistry and Biomedical Sciences<br>Goodfellow, Rhian; Cardiff University, School of Medicine<br>Vallender, Rebecca; Cardiff University<br>Chung, Ching-Wa; University of Aberdeen, School of Medicine, Medical Sciences and Nutrition<br>McKinley, Robert; Keele University, School of Medicine<br>Fuller, Richard; University of Liverpool Faculty of Health and Life Sciences, School of Medicine<br>Wong, Geoff; University of Oxford Division of Public Health and Primary Health Care, Nuffield Department of Primary Care Health Sciences

**Primary Subject Heading**:	Medical education and training
Secondary Subject Heading:	Qualitative research, Research methods
Keywords:	MEDICAL EDUCATION & TRAINING, QUALITATIVE RESEARCH, EDUCATION & TRAINING (see Medical Education & Training)

SCHOLARONE™
Manuscripts

TITLE PAGE:

a. Title of the article

Enhancing Authenticity, Diagnosticity and Equivalence (AD-Equiv) in multi-centre OSCE exams in Health Professionals Education. Protocol for a Complex Intervention Study

b. Full name, postal address, e-mail, telephone and fax numbers of the corresponding author

Peter Yeates, School of Medicine, David Weatherall Building, Keele University, Keele, ST5 5BG, UK, p.yeates@keele.ac.uk, Tel: +44 (0)1782 733930, Fax: +44 (0) 1782 733937.

c. Full names, departments, institutions, city and country of all co-authors:

Adriano Maluf, School of Medicine, Keele University, Newcastle-under-Lyme, UK;

Ruth Kinston, School of Medicine, Keele University, Newcastle-under-Lyme, UK;

Natalie Cope, School of Medicine, Keele University, Newcastle-under-Lyme, UK;

Gareth McCray, School of Medicine, Keele University, Newcastle-under-Lyme, UK;

Kathy Cullen, School of Medicine, Dentistry and Biomedical Sciences, Queen's University Belfast, Belfast, UK;

Vikki O'Neill, School of Medicine, Dentistry and Biomedical Sciences, Queen's University Belfast, Belfast, UK;

Aidan Cole, School of Medicine, Dentistry and Biomedical Sciences, Queen's University Belfast, Belfast, UK;

Rhian Goodfellow, School of Medicine, Cardiff University, Cardiff, UK;

Rebecca Vallander, School of Medicine, Cardiff University, Cardiff, UK;

Ching-Wa Chung, School of Medicine, University of Aberdeen, Aberdeen, UK;

Robert K McKinley, School of Medicine, Keele University, Newcastle-under-Lyme, UK;

Richard Fuller, School of Medicine, University of Liverpool, Liverpool, UK

Geoff Wong, Nuffield Department of Primary Care Health Sciences, University of Oxford, Oxford, UK.

d. Up to five keywords or phrases suitable for use in an index (it is recommended to use MeSH:

MEDICAL EDUCATION & TRAINING, QUALITATIVE RESEARCH, EDUCATION & TRAINING (see Medical Education & Training).

e. Word count - excluding title page, abstract, references, figures and tables: 4857 words

f. Number of Reference (Please supply the number of references, abstract count and word count in the title page.):

References: 49 references.

Abstract: 290 words.

Title page: 292 words.

For peer review only

Enhancing Authenticity, Diagnosticity and Equivalence (AD-Equiv) in multi-centre OSCE exams in Health Professionals Education. Protocol for a Complex Intervention Study

Peter Yeates, Adriano Maluf, Ruth Kinston, Natalie Cope, Gareth McCray, Kathy Cullen, Vikki O'Neill, Aidan Cole, Rhian Goodfellow, Rebecca Vallander, Ching-Wa Chung, Robert K McKinley, Richard Fuller, Geoff Wong.

Correspondence to Dr Peter Yeates; p.yeates@keele.ac.uk

For peer review only

Abstract

Introduction Objective structured clinical exams (OSCEs) are a cornerstone of assessing the competence of trainee healthcare professionals, but have been criticised for a/ lacking authenticity, b/ variability in examiners' judgements which can challenge assessment equivalence and c/ for limited diagnosticity of trainees' focal strengths and weaknesses. In response, this study aims to investigate whether a/ sharing integrated-task OSCE stations across institutions can increase perceived authenticity, whilst b/ enhancing assessment equivalence by enabling comparison of the standard of examiners' judgements between institutions using a novel methodology (VESCA) and c/ exploring the potential to develop more diagnostic signals from data on students' performances.

Methods and Analysis The study will use a complex intervention design, developing, implementing and sharing an integrated-task (research) OSCE across 4 UK medical schools. It will use "Video-based Score Comparison and Adjustment" (VESCA) to compare examiner scoring differences between groups of examiners and different sites, whilst studying how, why and for whom the shared OSCE and VESCA operates across participating schools. Quantitative analysis will use Many Facet Rasch Modelling to compare the influence of different examiners groups and sites on students' scores, whilst the operation of the two interventions (shared integrated task OSCEs; VESCA) will be studied

through the theory-driven method of Realist evaluation. Further exploratory analyses will examine diagnostic performance signals within data.

Ethics and Dissemination The study will be extra to usual course requirements and all participation will be voluntary. We will uphold principles of informed consent, the right to withdraw, confidentiality with pseudonymity and strict data security. The study has received ethical approval from Keele University Research Ethics Committee. Findings will be academically published and will contribute to good practice guidance on 1/ the use of VESCA and 2/ sharing and use of integrated-task OSCE stations.

For peer review only

Strengths and Limitations

- The study uses a complex intervention design to explain how two separate interventions operate when jointly shared across medical schools to address authenticity and equivalence: a/ integrated-task OSCE stations and b/ video-based examiner score comparison and adjustment (VESCA).
- The study's multi-centre design provides broadly sampled insight into the operation of integrated-task OSCE stations across different contexts
- Use of Realist Evaluation will give rich insight into how these interventions work or don't work, under what circumstances, for whom and why.
- Whilst it is part of the object of study to explore how institutional differences in implementation might alter OSCE conditions, any such effects could potentially bias estimates of examiner-cohort effects in the main analysis. This is a limitation. The study's use of video-based comparison of examiners' scoring will enable controlled comparison of a subset of these responses, which will also be presented to enable the likelihood of such bias to be judged.

Introduction

Dependable assessment of the performance and skills of graduating health professionals (doctors, nurses, physiotherapists, pharmacists etc) remains critical to ensuring fairness for students(1) and patient safety(2,3). OSCEs generally involve students rotating around a carousel of timed, simulated clinical tasks being observed on each task by different, trained, examiners who score performances using specified criteria (4). Over recent decades, Objective Structured Clinical Exams (OSCEs) have become one of the pre-eminent methods of assessing clinical skills performance(5) due to their ability to ensure students are directly observed (6) under equivalent conditions (7) according to an appropriate assessment blueprint(8) whilst avoiding some of the limitations of workplace assessments such as case selection, impression management(9), or prior performance information(10).

Despite these benefits, OSCEs have been criticised for:

- Lacking authenticity
- Examiner variability, which can challenge equivalence
- Limited ability to ensure that students are competent in all skills domains

The authenticity of OSCEs has been criticised due to their simulated context and task fragmentation (11,12), which in turn could challenge the applicability of their outcomes to clinical practice(13). In response, several institutions have explored use of OSCE stations which combine multiple tasks (14,15) – termed “integrated task OSCEs” or greater levels of simulation fidelity (16) to more closely mimic real practice. Whilst these appear to offer a promising development, it is unclear how the added complexity of these tasks influences examiners’ judgements and therefore OSCE standardisation.

For peer review only

Furthermore, examiner variability in OSCEs continues to be significant(17). Owing to student numbers, OSCE exams are often run across several ostensibly identical parallel versions of the same exam or distributed across geographical locations, with different examiners in each parallel version. Several studies suggest potentially important differences between the different cohorts of examiners in each parallel version of the exam within single institutions(18) or in large scale distributed exams (19,20). Whilst these variations could compromise the fairness or safety of the resulting assessment decisions, they are rarely studied due to difficulties in directly measuring the influence of unlinked groups of examiners in different parallel versions of the exam. Consequently, little is known about how regional variations in examiners' judgements might challenge the equivalence of OSCEs (21) which could produce different outcomes for students in OSCE exams.

Two pre-requisites are necessary to determine equivalence within a distributed OSCE: firstly, common (or shared) OSCE content is needed, in order for examiners' judgements to be comparable, and secondly, a method is needed to compare examiners' scoring when they are distributed across different locations. In the UK, medical schools set their own OSCE exams, resulting in variation in content and format between Schools. Consequently, sharing OSCE content between schools, whilst necessary, will involve change from usual practice which could further influence examiner variability or produce unintended consequences.

Recently, Yeates et al (22–24) have iteratively developed a method to compare examiners' scoring within distributed OSCEs, called Video-based Examiner Score Comparison and Adjustment (VESCA). This produces linking of otherwise unlinked groups of examiners (termed "examiner-cohorts"(18) by 1/ videoing a small subset of students on each station of the OSCE; 2/ asking examiners from all examiner-cohorts to score the same station-specific comparator videos; and 3/ using the resulting score linkage to compare and equate for differences in examiner-cohorts. Their findings suggest that despite following accepted procedures for OSCE conduct, significant differences may persist between groups of examiners which could affect the pass/fail classification of a significant minority

For peer review only

of students. Follow-up work has enhanced the technique's feasibility (24), and shown that it is adequately robust to several potential confounding influences (25) and variations in implementation (26). Whilst these findings suggest that examiner-cohort effects are important and support the validity of VESCA for their measurement, VESCA has not yet been used across institutions, so both the likely magnitude of effects which may arise, and the practical implications of applying the method across institutions are unknown.

Finally, recent inquiry has focused on ensuring that trainees are competent across all relevant domains of performance(27), with a view to both providing diagnostic information to support their learning and enabling focused areas of deficit to be addressed rather than simply demonstrating a sufficient total score, as is often the case in OSCEs (28). This had led to scrutiny of the ability of OSCEs to prevent compensation between domains (29) and whether OSCEs could provide greater diagnosticity of students' areas of focal weakness. Whilst non-compensatory domain-based scoring has been trialled in other arenas (30), little is known about the psychometric properties of such domain scores or whether they can provide independent reliable scores for the constructs they represent. As the utility of VESCA would be greatly enhanced by providing domain level information which has been adjusted for the examiner-cohort effects, it is desirable to study the potential for these data to provide that information.

Collectively, it is anticipated that if these interventions are able to enhance the authenticity and equivalence of OSCEs whilst providing more diagnostic information on learners' performance, this will enhance OSCEs ability to support learning through their influence on students' preparation for OSCEs and their subsequent provision of more diagnostic feedback, whilst also ensuring greater confidence in the progression decisions which they inform. Consequently, understanding the interaction and use of these innovations is critical to determining their ability to benefit educational and healthcare practice.

For peer review only

Aims and Objectives

This project has a series of aims, objectives and research questions that set out to address the criticisms described above about OSCE examinations. These are:

Criticism 1: Lack of authenticity

- Objective 1: to increase perceived authenticity of an OSCE through use of integrated-task OSCE stations

Criticism 2: Examiner variability and challenges to equivalence

- Objective 2: to share integrated-task OSCE stations across different institutions and understand the implications which arise from the interaction of these stations with existing individual perceptions and institutional assessment practices.

Then, developing from that objective

- Objective 3: to use the VESCA methodology, within the context of a multi-centre integrated-task OSCE, to
    a. compare and equate for differences between examiner-cohorts in different institutions and
    b. understand the implications which arise from using VESCA across institutions.

Criticism 3: Limited diagnosticity of OSCEs across different domains of performance

- Objective 4: to determine whether different sub-domains of performance can be reliably distinguished from each other (rather than only providing an overall competence score) within a shared integrated-task OSCE.

For peer review only

Research Questions

Objectives 1 and 2 will be addressed jointly through research question 1

When integrated-task stations are used and shared within an OSCE, how, when, why and to what extent do examiners, students and simulated patients use and interact with them and how does this influence their perception of the authenticity of the OSCE scenarios?

Objective 3a will be addressed by the following research questions 2-5

2. How does the standard of examiners' judgements compare between examiner-cohorts?
3. How does the standard of examiners' judgements compare between institutions?
4. What are the relative magnitudes of inter versus intra institutional variation?
5. How much influence does adjusting for examiner-cohort effects have on students':
    a. Overall Scores
    b. Categorisation (fail / pass / excellence)
    c. Rank position

Objective 3b will be addressed through research question 6

When VESCA is used to compare and equate for differences between examiner-cohorts in different institutions within the context of a shared integrated-task OSCE, how, when, why and to what extent do examiners, students and simulated patients use and interact with VESCA?

Objective 4 will be addressed through research questions 7-8

7. How reliably can different domains of assessment be discriminated in unadjusted data?
8. Do students show differing patterns of performance across different domains of the assessment in unadjusted data?

For peer review only

Methods

Methodological Overview

The study will use a complex intervention design(31) to implement Video-based Examiner Score Comparison and Adjustment (VESCA) in the context of a multi-centre authentic-task OSCE. Research approaches will comprise psychometric analysis of assessment data(32) and Realist evaluation(33), collecting data through mixed methods. A schematic overview of the data collection and analysis is provided in figure 1.

Population, Sampling and Recruitment

The study population will comprise participants of late years (penultimate and final year) undergraduate medical student clinical exams within the United Kingdom.

This population will be sampled by recruiting four medical schools to participate as centres in the study, with sampling from all relevant examiners, students, simulated patients. As no prior work has formally compared OSCE examination standards across UK medical schools, the study will aim to sample across different characteristics which might plausibly influence scoring: geographic divergence; Russell group and non-Russell group Universities; and new and more established medical schools.

Recruitment will be performed locally by each participating institution using both in-person and electronic advertisements. Each participating institute will have recruitment targets for students (n=24), examiners (n=12), and simulated patients (n=12). This sample size is pragmatic based on the resource implications for individual institutions of running a research OSCE. Whilst no formal method exists to power comparisons, or any agreed minimally important difference for differences between groups of OSCE examiners, subset analysis of data from Yeates et al 2021(24) suggests this

For peer review only

sample size is likely to provide a standard error in the region of 0.03 logits, enabling statistically significant detection of a difference between examiner cohorts of 5% of the assessment scale.

OSCE Design

The OSCE will comprise six tasks (stations). In each station, students will be directly observed for 13.5 minutes, with a further variable amount of preparation and rotation time of between 1.5 – 4 minutes per station, depending on each school's usual practice. Consequently, total testing time will range between 90-105 mins depending on different schools' practices.

Station content (simulated patient scenarios / instructions / stimulus materials / scoring rubrics) will be developed by the research team to reflect plausible simulated scenarios from Foundation year 1 doctors routine work and integrate multiple related processes which would be required for whole-task completion. For example, a station may describe a specific clinical scenario from the work of a new doctor and instruct candidates to perform a relevant clinical assessment. Candidates might then be expected to gather a clinical history, perform relevant focused physical examination, interpret provided investigation results, consult available guidelines and then describe their diagnosis and management to the patient. Tasks will be blueprinted against the UK General Medical Council's Clinical Skills Performance Assessment framework(34), to sample this framework's 3 domains: areas of clinical practice; clinical and professional capabilities; and areas of professional knowledge. The same stations will be used in all 4 study sites, whilst allowing minor adaptation for local contexts (for example by providing local antibiotic guidelines or dosage calculators).

Individual students will rotate around all 6 stations, and be observed by a different, single examiner in each station during a 90 minute "cycle" of the exam. Each site will host two parallel circuits of the OSCE (identical OSCE stations, run with different examiners). Twelve students will be examined in each parallel circuit (i.e. two cycles of 6 students), enabling 24 students to be tested at each site.

For peer review only

Examiners will be provided with station material (clinical scenario, simulated patient script, marking criteria) prior to the OSCE. Additionally, examiners will be provided with a web-link to a training video which will orientate them to the scoring format.

Examiners at all sites will score students' performances on the GeCoS rating system(35). This scoring system selects 5 appropriate performance domains for each station from a list of 20 when the station is designed (for example: history content, physical examination, clinical reasoning, building and maintaining the relationship, management content). Each domain is scored 1-4 (1=must improve; 2=borderline; 3=proficient; 4=very good). These scores are combined with a further 7-point global rating (1=incompetent; 7=excellent) to give a total score out of 27 for each station. Scoring will use tablet or paper-based marking based on available resources at each site.

The OSCE will be conducted first at the lead site (Keele) to enable video production for VESCA procedures; timing in other institutions will vary within an 8 month window to fit with local curricular demands. Local site teams will operationalise the station content based on the constraints of their local resources and equipment. Timing of stations will use local timing facilities but will adhere to standard timing intervals.

Intervention

VESCA will be employed using the methods developed by Yeates et al (22–24).

Video filming: Performances of all students in all 6 stations, from the first cycle, on a selected circuit, will be filmed at the lead site (Keele) using methods established by Yeates et al (23). Filming will use two unobtrusive wall-mounted closed-circuit TV cameras in every room (ReoLink 432, 1080 HD resolution). Camera position, angle and zoom will be selected to optimise capture of the performance. Sound will be recorded using a stereo condensing boundary microphones (Audio-Technica Pro 44). The first three videos from each station which are technically adequate

For peer review only

(unobstructed pictures with adequate sound) will be selected and processed for further use, resulting in three comparison videos for each of the six stations in the OSCE.

Video scoring: All examiners will be asked to score the three selected videos selected for the station they examine. All examiners who examine a given station will score the same videos. To facilitate this, videos will be securely shared across institutions, using the secure on-line video scoring approach developed by Yeates et al(24). This will include the following elements: on-line consent; station-specific examiner information; sequential presentation of the 3 comparison videos for the station. Examiners will have to score each video and provide brief feedback before progressing to the next. As per Yeates et al (23), examiners will have 4 weeks after the OSCE to complete video scoring.

Data Collection

Student scores (live and video performances) from each site will be collated and labelled with unique identifiers indicating 1/ student, 2/ site, 3/ circuit, 4/ station, 5/examiner, 6/ examiner-cohort and 7/ video or live performance. These data will be used to address all psychometric research questions.

To address research questions 1 & 6, researchers will develop an initial programme theory (IPT)(36) to orientate and focus subsequent data collection and analysis. To develop the initial programme theory, researchers will consider prior research on VESCA, published experiences of international multi-institutional OSCE collaborations, formal theories which concern institutional adoption of innovations, and the views of a range of experience assessment professionals.

Data will be collected iteratively, interspersed by analysis(37), through a mixture of observation, individual interviews (38) and (where feasible) focus groups, supplemented by available process data. This, along with score data, will be triangulated across modalities to support validity.

Interviews will sample individuals from all relevant stakeholder groups at each site, focused on individuals who have participated in the research OSCE. Whilst sampling requirements will be data

For peer review only

driven, indicative numbers of each group from each site are students (n=4), examiners (n=4), simulated patients(n=3), and OSCE administrators(n=1-2). All individuals participating in the OSCE will be invited to be interviewed. If offers of participation exceed sampling needs, then participants will be selected to maximise sample representativeness. Recruitment will be performed by email. Participation will be voluntary. Participants will receive study information and asked to record their consent through an on-line consent form. Interviews will be conducted by members of the research team (PI, or research assistants), and are expected to last 45-60 mins. Interviews will be conducted in-person in a private place or via Microsoft Teams. Interviews will be audio recorded and professionally transcribed. Interviews will be guided by a topic guide which will draw from the IPT and evolving theory and will be illustrated by practice-based examples where needed. The interview approach will be adapted to glean, refine and then consolidate emerging theory (39).

Two researchers will observe the “on-the-day” conduct of the OSCE in each participating medical school, using Realist ethnographic observation methods (40). As far as feasible this will include: preparation for the OSCE, including station layout, equipment set-up, timing and scoring methods; conduct of the OSCE, including student flow around the circuits and observation of students examiners and simulated patients behaviour and interactions during and between station performances; students and examiners interaction with filming; and participants’ responses to both the OSCE and VESCA in breaks or after the OSCE is complete. Researchers’ observations will be recorded through field notes which may be supplemented by examples of items or materials from the OSCE, diagrams or photographs.

Process data will be collected by researchers from each school depending on availability and may include participant recruitment data, score data, website metrics from examiner training materials and metrics related to video scoring by examiners.

For peer review only

Patient and Public Involvement

Patients and members of the public have been involved throughout the VESCA programme of research which has led to this study. This has included establishing the priority of the research, reviewing plain English summaries, contributing to the design of the research, reviewing progress contributing to elements of the analysis and interpreting findings and discussing future directions. Members of the public are expected to contribute to dissemination activities.

Analysis

For peer review only

Realist Analyses (used for data relating to research questions 1 & 6)

Similar analysis methods will be used for both questions. Audio recordings of interviews and focus groups will be professionally transcribed. Observation field notes, where available, will be incorporated into the dataset as will summaries of score data, participation rates and engagement metrics from on-line video scoring by examiners and video access metrics from the on-line feedback portal for students. Analysis will use the stages described by Papoutsi et al (41). This begins by reading or considering each piece of data line-by-line to judge its relevance to the initial programme theory. Next, where needed, decisions will be made about the trustworthiness of relevant data. Next, researchers will allocate initial conceptual labels. Conceptual labels will be derived both deductively from the initial programme theory and inductively based on researchers' interpretation of emergent issues. Researchers will then consider whether each labelled concept can be interpreted to represent a context (C), a mechanism (M) or an outcome (O) and will look for data which provides information on the relatedness of Cs, Ms, and Os, so that they may be developed into Context-Mechanism-Outcome-Configurations (CMOC). Drawing on relevant data, researchers will then interpret how each CMOC relates to the programme theory and iteratively revise the programme theory as more and more CMOCs are developed. Interpretation will use the analytic processes of juxtaposition, reconciliation, adjudication and consolidation to explore discrepancies

and resolve differences. Interpretation will also use retro-duction, combining both induction based on emergence from the data and deduction from the initial programme theory in order to unearth mechanistic relations within CMOCs and the Programme theory(42,43). Analysis will proceed iteratively, interspersed with new data collection until a coherent and plausible programme theory is reached.

Psychometric analyses (used for RQs 2-5, 7,8)

Research questions 2-5 will be addressed using Many Facet Rasch Modelling (MFRM), conducted using FACETs by Winsteps (44). The dependent variable for analyses will be denoted "total score" and will be calculated for each student on each station by combining the scores for each domain. Categorical independent variables will be available for each station score, describing the student (unique ID number); station (station number); examiner (examiner ID); examiner-cohort (ex-cohort ID); and site (institution ID). These data will be analyses using a four facet Rasch model, with facets of: 1/ student, 2/ station, 3/ examiner-cohort and 4/ site.

To ensure data are adequate for MFRM analysis, research will assess the dimensionality, ordinality and model-fit of data. Dimensionality will be assessed using principle components analysis (PCA) of model residuals with random parallel analysis using R studio for R(45). Ordinality of the scale will be determined by examining the Rasch-Andrich thresholds supplied in FACETs output data(FACETS v3.82.3 Winsteps, Western Australia). Fit parameters supplied by FACETs will be examined to determine data to model fit, using the criteria advocated by Linacre (46). If data are inadequate for MFRM analysis, then the analysis plan will be adapted to use an appropriate alternative method such as linear mixed modelling.

To explore the potential that differences in institutional implementation of the OSCE might confound the measurement of examiner-cohort effects between institutions, we will additionally compare examiner cohort effects on the subset of score data arising from examiners' video scoring. This will offer a controlled comparison (as all examiner cohorts will score the same video

For peer review only

performances). Analysis will use generalised linear modelling (GLiM), including only data from examiners scoring of videos. The dependent variable will be total score, with factors of: station, examiner-cohort, and school will be included in the model. Results from this analysis will be presented alongside the main analysis, to enable the likelihood of bias in the MFRM to be judged as part of overall evaluation of the complex intervention.

To address RQ2, observed (Raw score) average scores and “Fair-Average” scores(47) for examiner-cohorts will be compared, and the difference between observed (Raw score) average and Fair average will be calculated for each examiner-cohort and compared. Observed differences will be transformed into multiples of the standard error to calculate statistical significance.

To address RQ3 observed (Raw score) average scores and “Fair-Average” scores(47) for each site (institution) will be compared and the difference between their observed (Raw score) average and Fair average will be calculated for each site and compared

To address RQ4, the difference between examiner-cohorts within each institution (i.e. site) will be calculated and compared with the differences between the values for different institutions

To address RQ5a, the difference between the raw observed average score and the fair average score will be calculated for each participating student. These will be converted to mean absolute differences (MAD) to remove the direction of score adjustment. Descriptive statistics will be calculated for both the raw score adjustments and MAD adjustments. Similar to prior research (22,24), the effect size of each MAD score adjustment will calculated using Cohen’s d (48), using the standard deviation of students’ average observed scores as the denominator. The mean Cohen’s d and the proportion of students’ whose adjustment exceeds d=0.5 will be reported.

To address RQ5b&c, category boundaries will be developed using the borderline regression method(49) for each station and pooled to give an average cut score for the test. Two separate values will be interpolated from the x-axis: one to represent a fail/pass boundary and one to

For peer review only

represent a pass/excellent boundary. Each students' categorisation for the OSCE relative to these boundaries will be determined based on their observed raw average score and their fair average score and the proportion changing categories (number increasing a grade; number reducing a grade) will be calculated for both thresholds. Students rank position in the OSCE (regardless of institutional rankings) will be calculated based on observed raw average scores and fair average scores and the difference between each student's rank position from each score calculated. This will be expressed as both raw change in rank (positive or negative sign) and MAD change in rank which will be summarised through descriptive statistics.

Research questions 7&8 represent exploratory forms of analysis. These analyses will use the scores in individual scores domains within each station as dependent variables. Domains will be grouped based on content into dimensions which represent communication skills, knowledge and reasoning, investigation and management and procedural skills. Exploratory Factor Analysis will be used to determine the level of support for these dimensions, and Cronbach's alpha will be used to estimate the reliability of scores within each dimension. Student-level dimension scores will be examined to produce descriptive statistics describing dimension level scores and to determine the proportion of students who show greater than 0.5 standard deviation score difference between difference dimensions. Further exploratory analyses will determine whether categorical differences exist for some students across domains (i.e. greater frequency of borderline categories in 1 domain).

For peer review only

Anticipated Outcomes

Realist evaluations will produce mature programme theories which describe how different contexts elicit different mechanisms to produce varied outcomes for different stakeholders when a/ an integrated authentic task OSCE is shared between medical schools and b/. VESCA is implement across multiple medical schools. This will be used to produce guidance on successful implementation

of both interventions. Realist Evaluations will be reported using the standards of the RAMESES II reporting standards(38).

Psychometric analyses for RQs 2-5 will describe the extent of overall score variability which arose between examiner-cohorts and institutions in the standard of examiners' judgements, and the impact of adjustment for these on students' scores, categorisation and rank.

Psychometric analyses for RQs 7&8 will describe the dimensionality of domain-score data and varied patterns of strength and weakness in students' performances, with comparison in patterns across schools.

Outputs and Dissemination

Study reporting will describe the blue printing and station development process; scoring format; an overview of station content and test reliability.

Findings of the research will be disseminated through academic publications, conference presentations and workshops and through engagement meetings with educational institutions who may adopt or implement VESCA or Video-based feedback.

Outputs

Good practice guidelines for the use of VESCA to enhance OSCE examiner standardisation in distributed exams & for sharing integrated task OSCEs across institutions. Intended audiences: institutions, assessment leads, examiners. Engagement work through the Association for the Study of Medical Education Psychometrics Specialist Interest Group (ASME psychoSIG) to promote this to policy makers.

Explanatory video, describing the purpose, use and benefits of VESCA for a lay audience. Intended audience, students, examiners, members of the public.

Publications

For peer review only

The research is expected to produce academic publications describing the following findings:

1. Paper 1: primary psychometric analyses, comparing the influence of examiner-cohorts and institutions on students' scores, categorisation and rank
2. Paper 2: secondary psychometric analyses, determining the extent of additional diagnostic information available in domain score data.
3. Paper 3: Realist evaluation, a programme theory of the implications of using VESCA within a shared OSCE.

Ethical Considerations

This study will recruit volunteer students, examiners and SPs. Recruitment will invite the entirety of relevant students and examiner populations, subject to any local exclusions (for example adequate academic progress). Simulated patients will participate as per their usual professional working arrangements. Participants will retain the right to withdraw up until their data are anonymised after which point withdrawal will not be possible. Researchers will collect personal data to manage recruitment and to link scores from the OSCE, on-line usage and engagement data for video access or scoring and interview and focus group data. These data will be stored securely and treated as confidential. Access will be limited to those members of the research team who require access for the analyses specified within the research. Participants will be asked to indicate whether they permit their data to be used in future research or to be contacted about future research. There are few anticipated risks to participants: if videos, score or interview data pertaining to them were disseminated inadvertently then that could cause embarrassment or distress. This risk is mitigated through the confidentiality and data security measures which will be employed. Students may benefit from taking part in the research through the experience of novel OSCE assessment tasks or availability of video feedback. Examiners may benefit from practice at examining. Ethical approval for the study has been granted by Keele University Research Ethics committee (Ref: MH-210209)

For peer review only

Anticipated Timeframe

Developing collaborations: complete by end May 2021.

Finalising protocol: June 2021

Ethics application: July-Sept 2021

OSCE station development: September - October 2021

Scheduling and recruitment of OSCEs: October 2021 – March 2022

Site 1 OSCE: December – March 2022

Sites 2-4 OSCE: January – July 2022

Examiner video scoring: 4-week interval after each OSCE

Interviews / focus groups / observations: December – August 2022

Psychometric analyses: July – November 2022

Realist analysis February - November 2022

Dissemination: December 2022 - February 2023.

Contributorship

The study design was developed by PY, RK, NC, GM, KC, VO, AC, RG, RV, CWC, RKM and RF. PY wrote the original draft. GW provided expertise in Realist Evaluation methodology. PY, AM and NC are collecting data supported by KC, RV, CWC, RG, RV. PY and AM will analyse the data. All authors critiqued and provided edits to the manuscript for intellectual content.

For peer review only

Funding Statement

The study is funded through a National Institute for Health Research (NIHR) Clinician Scientist award held by the Principal Investigator, Peter Yeates. The study constitutes independent research and does not represent the views of the NIHR, the NHS or the department of health and social care.

Competing of Interests

There are no competing interests for any author.

References

1. Valentine N, Durning S, Shanahan EM, Schuwirth L. Fairness in human judgement in assessment: a hermeneutic literature review and conceptual framework. Adv Heal Sci Educ. 2020;26(2):713–38.

2. Eva KW. Cognitive Influences on Complex Performance Assessment: Lessons from the Interplay between Medicine and Psychology. J Appl Res Mem Cogn. 2018;7(2):177–88.

3. Kogan JR, Conforti LN, Iobst WF, Holmboe ES. Reconceptualizing variable rater assessments as both an educational and clinical care problem. Acad Med. 2014 May;89(5):721–7.

4. Newble D. Techniques for measuring clinical competence: objective structured clinical examinations. Med Educ. 2004;38:199–203.

5. Boursicot K, Kemp S, Wilkinson T, Findyartini A, Canning C, Cilliers F, et al. Performance assessment: Consensus statement and recommendations from the 2020 Ottawa Conference. Med Teach. 2021 Jan 2;43(1):58–67.

For peer review only

6. Harden RM, Stevenson M, Downie WW, Wilson GM. Medical Education Assessment of Clinical Competence using Objective Structured Examination. Br Med J. 1975;1(February):447–51.

7. Norcini J, Anderson MB, Bollela V, Burch V, Costa MJ, Duvivier R, et al. 2018 Consensus framework for good assessment. Med Teach. 2018;0(0):1–8.

8. Wass V, Vleuten C Van Der, Shatzer J, Jones R. Medical education quartet Assessment of clinical competence. Lancet. 2001;357:945–9.

9. Huffman BM, Hafferty FW, Bhagra A, Leasure EL, Santivasi WL, Sawatsky AP. Resident impression management within feedback conversations: A qualitative study. Med Educ. 2021;55(2):266–74.

10. Murto SH, Shaw T, Touchie C, Pugh D, Cowley L, Wood TJ. Are raters influenced by prior information about a learner ? A review of assimilation and contrast effects in assessment. Adv Heal Sci Educ. 2021;(0123456789).

11. Johnston JL, Kearney GP, Gormley GJ, Reid H. Into the uncanny valley: Simulation versus simulacrum? Med Educ. 2020;54(10):903–7.

12. Gormley GJ, Hodges BD, McNaughton N, Johnston JL. The show must go on? Patients, props and pedagogy in the theatre of the OSCE. Med Educ. 2016;50(12):1237–40.

13. Downing SM. Validity: on meaningful interpretation of assessment data. Med Educ. 2003 Sep;37(9):830–7.

14. Ruesseler M, Weinlich M, Byhahn C, Müller MP, Jünger J, Marzi I, et al. Increased authenticity in practical assessment using emergency case OSCE stations. Adv Heal Sci Educ. 2010;15(1):81–95.

15. Schoenmakers B, Wens J. The objective structured clinical examination revisited for

For peer review only

postgraduate trainees in general practice. Int J Med Educ. 2014;5:45–50.

16. Gormley G, Sterling M, Menary A, McKeown G. Keeping it real! Enhancing realism in standardised patient OSCE stations. Clin Teach. 2012;9(6):382–6.

17. Gingerich A. The Reliability of Rater Variability. J Grad Med Educ. 2020;12(2):159–61.

18. Yeates P, Sebok-Syer SS. Hawks, Doves and Rasch decisions: Understanding the influence of different cycles of an OSCE on students' scores using Many Facet Rasch Modeling. Med Teach. 2017;39(1):92–9.

19. Sebok SS, Roy M, Klinger D a, De Champlain AF. Examiners and content and site: Oh My! A national organization's investigation of score variation in large-scale performance assessments. Adv Health Sci Educ Theory Pract. 2015 Aug 28;20(3):581–94.

20. Floreck LM, De Champlain AF. Assessing Sources of Score Variability in a Multi-Site Medical Performance Assessment: An Application of Hierarchical Linear Modeling. Acad Med. 2001;76(10):S93–5.

21. Norcini J, Anderson B, Bollela V, Burch V, Costa MJ, Duvivier R, et al. Criteria for good assessment: consensus statement and recommendations from the Ottawa 2010 Conference. Med Teach. 2011 Jan;33(3):206–14.

22. Yeates P, Cope N, Hawarden A, Bradshaw H, McCray G, Homer M. Developing a video-based method to compare and adjust examiner effects in fully nested OSCEs. Med Educ. 2019 Mar;53(3):250–63.

23. Yeates P, Moult A, Lefroy J, Walsh-House J, Clews L, McKinley R, et al. Understanding and developing procedures for video-based assessment in medical education. Med Teach. 2020 Nov 1;42(11):1250–60.

24. Yeates P, Moult A, Cope N, McCray G, Xilas E, Lovelock T, et al. Measuring the Effect of

For peer review only

Examiner Variability in a Multiple-Circuit Objective Structured Clinical Examination (OSCE). Acad Med. 2021 Mar 2;96(8):1189–96.

25. Yeates P, Moult A, Cope N, McCray G, Fuller R, McKinley R. Determining influence, interaction and causality of contrast and sequence effects in objective structured clinical exams. Med Educ. 2022;56(3):292–302.

26. Yeates P, McCray G, Moult A, Cope N, Fuller R, McKinley R. Determining the influence of different linking patterns on the stability of students' score adjustments produced using Video-based Examiner Score Comparison and Adjustment (VESCA). BMC Med Educ. 2022;22(1):1–9.

27. Frank JR, Snell LS, Cate O Ten, Holmboe ES, Carraccio C, Swing SR, et al. Competency-based medical education: theory to practice. Med Teach. 2010 Aug 27;32(8):638–45.

28. McKinley DW, Norcini JJ. How to set standards on performance-based examinations: AMEE Guide No. 85. Med Teach. 2014;36(2):97–110.

29. Homer M, Russell J. Conjunctive standards in OSCEs: The why and the how of number of stations passed criteria. Med Teach. 2021;43(4):448–55.

30. Pearce J, Reid K, Chiavaroli N, Hyam D. Incorporating aspects of programmatic assessment into examinations: Aggregating rich information to inform decision-making. Med Teach. 2021 Feb 8;0(0):1–8.

31. Craig P, Dieppe P, Macintyre S, Michie S, Nazareth I, Petticrew M. Developing and evaluating complex interventions : new guidance. 2008.

32. Bond T, Fox C. Applying the Rasch Model Fundamental Measurement in the Human Sciences. 2nd Editio. New York & London: Routledge; 2012.

33. Pawson R, Tilley N. Realistic Evaluation. 1st ed. London: Sage Publications Ltd; 1997.

For peer review only

34. General Medical Council. MLA content map.

35. Lefroy J, Gay SP, Gibson S, Williams S, McKinley RK. Development and face validation of an instrument to assess and improve clinical consultation skills. Int J Clin Ski. 2011;5(2):115–125.

36. Pawson R, Manzano-Santaella A. A realist diagnostic workshop. Evaluation. 2012;18(2):176–91.

37. Marchal B, van Belle S, van Olmen J, Hoerée T, Kegels G. Is realist evaluation keeping its promise? A review of published empirical studies in the field of health systems research. Evaluation. 2012;18(2):192–212.

38. Wong G, Westhorp G, Manzano A, Greenhalgh J, Jagosh J, Greenhalgh T. RAMESES II reporting standards for realist evaluations. BMC Med. 2016;14(1):1–18.

39. Manzano A. The craft of interviewing in realist evaluation. Evaluation. 2016;22(3):342–60.

40. Hammersley M. Ethnography and Realism. In: Huberman AM, Miles MB, editors. The Qualitative Researcher's Companion. 2455 Teller Road, Thousand Oaks California 91320 United States of America: SAGE Publications, Inc.; 2002. p. 65–80.

41. Papoutsi C, Mattick K, Pearson M, Brennan N, Briscoe S, Wong G. Interventions to improve antimicrobial prescribing of doctors in training (IMPACT): a realist review. Heal Serv Deliv Res. 2018;6(10):1–136.

42. Astbury B, Leeuw FL. Unpacking Black Boxes: Mechanisms and Theory Building in Evaluation. Am J Eval. 2010;31(3):363–81.

43. The RAMESES II Project. Retroduction in realist evaluation. Nihr. 2017;(p 207):1–3.

44. Linacre JM. Many-Facet Rasch Measurement. 2nd Edicat. Chicago: MESA Press; 1994.

45. Team Rs. RStudio: Integrated Development for R. Boston, MA: RStudio, Inc.; 2015.

For peer review only

46. Linacre JM. What do Infit and Outfit, Mean-square and Standardized mean? [Internet]. Rasch.Org website. 2002 [cited 2018 Jun 12]. p. 16:2, p878. Available from: https://www.rasch.org/rmt/rmt162f.htm

47. Linacre JM. A User's guide to FACETS Rasch-Model Computer Programs. 2005.

48. Cohen J. Statistical Power Analysis for the Social Sciences. 2nd ed. Lawrence Erlbaum Associates; 1988.

49. Downing SM, Tekian A, Yudkowsky R. Procedures for Establishing Defensible Absolute Passing Scores on Performance Examinations in Health Professions Education Procedures for Establishing Defensible Absolute Passing Scores on Performan. Teach Learn Med. 2006;18(1):50–7.

Figure 1: Schematic of the data collection and analysis processes

For peer review only

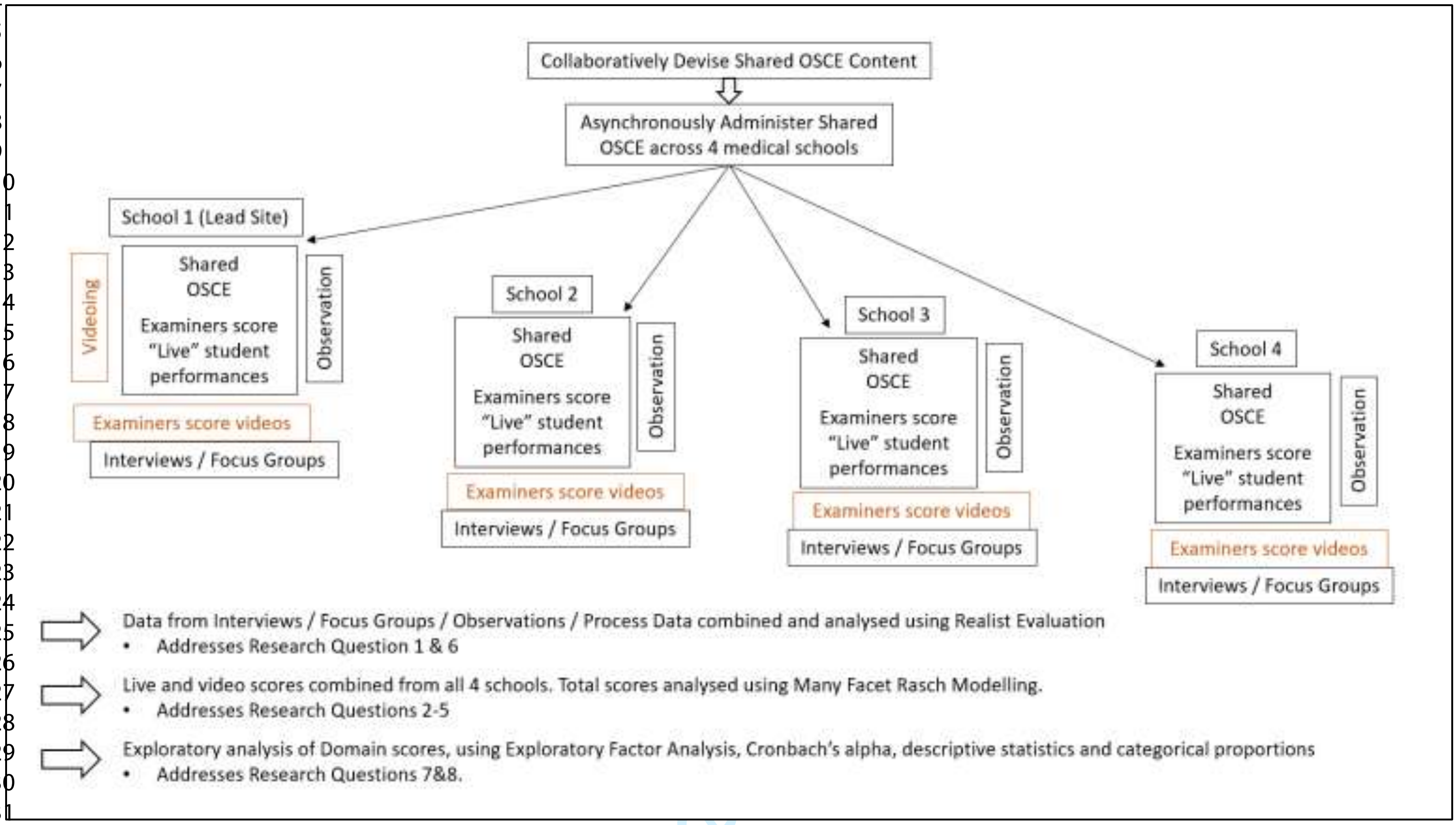
Collaboratively Devise Shared OSCE Content
Asynchronously Administer Shared OSCE across 4 medical schools
School 1 (Lead Site)
Videoing
Shared OSCE
Examiners score "Live" student performances
Observation
Examiners score videos
Interviews / Focus Groups
School 2
Shared OSCE
Examiners score "Live" student performances
Observation
Examiners score videos
Interviews / Focus Groups
School 3
Shared OSCE
Examiners score "Live" student performances
Observation
Examiners score videos
Interviews / Focus Groups
School 4
Shared OSCE
Examiners score "Live" student performances
Observation
Examiners score videos
Interviews / Focus Groups
Data from Interviews / Focus Groups / Observations / Process Data combined and analysed using Realist Evaluation
• Addresses Research Question 1 & 6
Live and video scores combined from all 4 schools. Total scores analysed using Many Facet Rasch Modelling.
• Addresses Research Questions 2-5
Exploratory analysis of Domain scores, using Exploratory Factor Analysis, Cronbach's alpha, descriptive statistics and categorical proportions
• Addresses Research Questions 7&8.

CONSORT 2010 checklist of information to include when reporting a randomised trial*

Section/Topic	Item No	Checklist item	Reported on page No
**Title and abstract**			
	1a	Identification as a randomised trial in the title	n/a
	1b	Structured summary of trial design, methods, results, and conclusions (for specific guidance see CONSORT for abstracts)	1-2
**Introduction**			
Background and objectives	2a	Scientific background and explanation of rationale	3-5
	2b	Specific objectives or hypotheses	5-7
**Methods**			
Trial design	3a	Description of trial design (such as parallel, factorial) including allocation ratio	7-10
	3b	Important changes to methods after trial commencement (such as eligibility criteria), with reasons	n/a
Participants	4a	Eligibility criteria for participants	10
	4b	Settings and locations where the data were collected	10
Interventions	5	The interventions for each group with sufficient details to allow replication, including how and when they were actually administered	10-11
Outcomes	6a	Completely defined pre-specified primary and secondary outcome measures, including how and when they were assessed	n/a
	6b	Any changes to trial outcomes after the trial commenced, with reasons	n/a
Sample size	7a	How sample size was determined	10-11
	7b	When applicable, explanation of any interim analyses and stopping guidelines	n/a
Randomisation:			
Sequence generation	8a	Method used to generate the random allocation sequence	n/a
	8b	Type of randomisation; details of any restriction (such as blocking and block size)	n/a
Allocation concealment mechanism	9	Mechanism used to implement the random allocation sequence (such as sequentially numbered containers), describing any steps taken to conceal the sequence until interventions were assigned	n/a
Implementation	10	Who generated the random allocation sequence, who enrolled participants, and who assigned participants to interventions	n/a
Blinding	11a	If done, who was blinded after assignment to interventions (for example, participants, care providers, those	n/a

For peer review only

For peer review only - http://bmjopen.bmj.com/site/about/guidelines.xhtml

		assessing outcomes) and how	
	11b	If relevant, description of the similarity of interventions	4
Statistical methods	12a	Statistical methods used to compare groups for primary and secondary outcomes	12-14
	12b	Methods for additional analyses, such as subgroup analyses and adjusted analyses	11-12
**Results**			
Participant flow (a diagram is strongly recommended)	13a	For each group, the numbers of participants who were randomly assigned, received intended treatment, and were analysed for the primary outcome	n/a
	13b	For each group, losses and exclusions after randomisation, together with reasons	n/a
Recruitment	14a	Dates defining the periods of recruitment and follow-up	17
	14b	Why the trial ended or was stopped	n/a
Baseline data	15	A table showing baseline demographic and clinical characteristics for each group	n/a
Numbers analysed	16	For each group, number of participants (denominator) included in each analysis and whether the analysis was by original assigned groups	n/a
Outcomes and estimation	17a	For each primary and secondary outcome, results for each group, and the estimated effect size and its precision (such as 95% confidence interval)	n/a
	17b	For binary outcomes, presentation of both absolute and relative effect sizes is recommended	n/a
Ancillary analyses	18	Results of any other analyses performed, including subgroup analyses and adjusted analyses, distinguishing pre-specified from exploratory	15-16
Harms	19	All important harms or unintended effects in each group (for specific guidance see CONSORT for harms)	16
**Discussion**			
Limitations	20	Trial limitations, addressing sources of potential bias, imprecision, and, if relevant, multiplicity of analyses	n/a
Generalisability	21	Generalisability (external validity, applicability) of the trial findings	15
Interpretation	22	Interpretation consistent with results, balancing benefits and harms, and considering other relevant evidence	11-14
**Other information**			
Registration	23	Registration number and name of trial registry	n/a
Protocol	24	Where the full trial protocol can be accessed, if available	n/a
Funding	25	Sources of funding and other support (such as supply of drugs), role of funders	22

*We strongly recommend reading this statement in conjunction with the CONSORT 2010 Explanation and Elaboration for important clarifications on all the items. If relevant, we also recommend reading CONSORT extensions for cluster randomised trials, non-inferiority and equivalence trials, non-pharmacological treatments, herbal interventions, and pragmatic trials. Additional extensions are forthcoming: for those and for up to date references relevant to this checklist, see www.consort-statement.org.11-114

For peer review only - http://bmjopen.bmj.com/site/about/guidelines.xhtml

BMJ Open

Enhancing Authenticity, Diagnosticity and Equivalence (AD-Equiv) in multi-centre OSCE exams in Health Professionals Education. Protocol for a Complex Intervention Study

Journal:	*BMJ Open*
Manuscript ID	bmjopen-2022-064387.R2
Article Type:	Protocol
Date Submitted by the Author:	11-Oct-2022

Complete List of Authors:	Yeates, Peter; Keele University, School of Medicine<br>Maluf, Adriano; Keele University, School of Medicine<br>Kinston, Ruth; Keele University, School of Medicine<br>Cope, Natalie; Keele University, School of Medicine<br>McCray, Gareth; Keele University, School of Primary, Community and Social Care<br>Cullen, Kathy; Queen's University Belfast, School of Medicine, Dentistry and Biomedical Sciences<br>O'Neill, Vikki; Queen's University Belfast, School of Medicine, Dentistry and Biomedical Sciences<br>Cole, Aidan; Queen's University Belfast, School of Medicine, Dentistry and Biomedical Sciences<br>Goodfellow, Rhian; Cardiff University, School of Medicine<br>Vallender, Rebecca; Cardiff University<br>Chung, Ching-Wa; University of Aberdeen, School of Medicine, Medical Sciences and Nutrition<br>McKinley, Robert; Keele University, School of Medicine<br>Fuller, Richard; University of Liverpool Faculty of Health and Life Sciences, School of Medicine<br>Wong, Geoff; University of Oxford Division of Public Health and Primary Health Care, Nuffield Department of Primary Care Health Sciences

**Primary Subject Heading**:	Medical education and training
Secondary Subject Heading:	Qualitative research, Research methods
Keywords:	MEDICAL EDUCATION & TRAINING, QUALITATIVE RESEARCH, EDUCATION & TRAINING (see Medical Education & Training)

SCHOLARONE™ Manuscripts

TITLE PAGE:

a. Title of the article

Enhancing Authenticity, Diagnosticity and Equivalence (AD-Equiv) in multi-centre OSCE exams in Health Professionals Education. Protocol for a Complex Intervention Study

b. Full name, postal address, e-mail, telephone and fax numbers of the corresponding author

Peter Yeates, School of Medicine, David Weatherall Building, Keele University, Keele, ST5 5BG, UK, p.yeates@keele.ac.uk, Tel: +44 (0)1782 733930, Fax: +44 (0) 1782 733937.

c. Full names, departments, institutions, city and country of all co-authors:

Peter Yeates, School of Medicine, Keele University, Newcastle-under-Lyme, UK;

Adriano Maluf, School of Medicine, Keele University, Newcastle-under-Lyme, UK;

Ruth Kinston, School of Medicine, Keele University, Newcastle-under-Lyme, UK;

Natalie Cope, School of Medicine, Keele University, Newcastle-under-Lyme, UK;

Gareth McCray, School of Medicine, Keele University, Newcastle-under-Lyme, UK;

Kathy Cullen, School of Medicine, Dentistry and Biomedical Sciences, Queen's University Belfast, Belfast, UK;

Vikki O'Neill, School of Medicine, Dentistry and Biomedical Sciences, Queen's University Belfast, Belfast, UK;

Aidan Cole, School of Medicine, Dentistry and Biomedical Sciences, Queen's University Belfast, Belfast, UK;

Rhian Goodfellow, School of Medicine, Cardiff University, Cardiff, UK;

Rebecca Vallender, School of Medicine, Cardiff University, Cardiff, UK;

Ching-Wa Chung, School of Medicine, University of Aberdeen, Aberdeen, UK;

Robert K McKinley, School of Medicine, Keele University, Newcastle-under-Lyme, UK;

Richard Fuller, School of Medicine, University of Liverpool, Liverpool, UK

Geoff Wong, Nuffield Department of Primary Care Health Sciences, University of Oxford, Oxford, UK.

d. Up to five keywords or phrases suitable for use in an index (it is recommended to use MeSH:

MEDICAL EDUCATION & TRAINING, QUALITATIVE RESEARCH, EDUCATION & TRAINING (see Medical Education & Training).

e. Word count - excluding title page, abstract, references, figures and tables: 4828 words

f. Number of Reference (Please supply the number of references, abstract count and word count in the title page.):

References: 49 references.

Abstract: 290 words.

Title page: 292 words.

For peer review only

Enhancing Authenticity, Diagnosticity and Equivalence (AD-Equiv) in multi-centre OSCE exams in Health Professionals Education. Protocol for a Complex Intervention Study

Peter Yeates, Adriano Maluf, Ruth Kinston, Natalie Cope, Gareth McCray, Kathy Cullen, Vikki O'Neill, Aidan Cole, Rhian Goodfellow, Rebecca Vallander, Ching-Wa Chung, Robert K McKinley, Richard Fuller, Geoff Wong.

Correspondence to Dr Peter Yeates; p.yeates@keele.ac.uk

For peer review only

Abstract

Introduction Objective structured clinical exams (OSCEs) are a cornerstone of assessing the competence of trainee healthcare professionals, but have been criticised for a/ lacking authenticity, b/ variability in examiners' judgements which can challenge assessment equivalence and c/ for limited diagnosticity of trainees' focal strengths and weaknesses. In response, this study aims to investigate whether a/ sharing integrated-task OSCE stations across institutions can increase perceived authenticity, whilst b/ enhancing assessment equivalence by enabling comparison of the standard of examiners' judgements between institutions using a novel methodology (VESCA) and c/ exploring the potential to develop more diagnostic signals from data on students' performances.

Methods and Analysis The study will use a complex intervention design, developing, implementing and sharing an integrated-task (research) OSCE across 4 UK medical schools. It will use "Video-based Score Comparison and Adjustment" (VESCA) to compare examiner scoring differences between groups of examiners and different sites, whilst studying how, why and for whom the shared OSCE and VESCA operates across participating schools. Quantitative analysis will use Many Facet Rasch Modelling to compare the influence of different examiners groups and sites on students' scores, whilst the operation of the two interventions (shared integrated task OSCEs; VESCA) will be studied

through the theory-driven method of Realist evaluation. Further exploratory analyses will examine diagnostic performance signals within data.

Ethics and Dissemination The study will be extra to usual course requirements and all participation will be voluntary. We will uphold principles of informed consent, the right to withdraw, confidentiality with pseudonymity and strict data security. The study has received ethical approval from Keele University Research Ethics Committee. Findings will be academically published and will contribute to good practice guidance on 1/ the use of VESCA and 2/ sharing and use of integrated-task OSCE stations.

For peer review only

Strengths and Limitations

- The study uses a complex intervention design to explain how two separate interventions operate when jointly shared across medical schools to address authenticity and equivalence: a/ integrated-task OSCE stations and b/ video-based examiner score comparison and adjustment (VESCA).
- The study's multi-centre design provides broadly sampled insight into the operation of integrated-task OSCE stations across different contexts
- Use of Realist Evaluation will give rich insight into how these interventions work or don't work, under what circumstances, for whom and why.
- Video-based comparison of examiners' scoring will provide controlled comparisons between schools of a subset of examiners' scoring, thereby enabling appraisal of the likelihood of bias arising from inter-institutional differences in implementation.

Introduction

Dependable assessment of the performance and skills of graduating health professionals (doctors, nurses, physiotherapists, pharmacists etc) remains critical to ensuring fairness for students(1) and patient safety(2,3). OSCEs generally involve students rotating around a carousel of timed, simulated clinical tasks being observed on each task by different, trained, examiners who score performances using specified criteria (4). Over recent decades, Objective Structured Clinical Exams (OSCEs) have become one of the pre-eminent methods of assessing clinical skills performance(5) due to their ability to ensure students are directly observed (6) under equivalent conditions (7) according to an appropriate assessment blueprint(8) whilst avoiding some of the limitations of workplace assessments such as case selection, impression management(9), or prior performance information(10).

Despite these benefits, OSCEs have been criticised for:

- Lacking authenticity
- Examiner variability, which can challenge equivalence
- Limited ability to ensure that students are competent in all skills domains

The authenticity of OSCEs has been criticised due to their simulated context and task fragmentation (11,12), which in turn could challenge the applicability of their outcomes to clinical practice(13). In response, several institutions have explored use of OSCE stations which combine multiple tasks (14,15) – termed “integrated task OSCEs” or greater levels of simulation fidelity (16) to more closely mimic real practice. Whilst these appear to offer a promising development, it is unclear how the added complexity of these tasks influences examiners’ judgements and therefore OSCE standardisation.

For peer review only

Furthermore, examiner variability in OSCEs continues to be significant(17). Owing to student numbers, OSCE exams are often run across several ostensibly identical parallel versions of the same exam or distributed across geographical locations, with different examiners in each parallel version. Several studies suggest potentially important differences between the different cohorts of examiners in each parallel version of the exam within single institutions(18) or in large scale distributed exams (19,20). Whilst these variations could compromise the fairness or safety of the resulting assessment decisions, they are rarely studied due to difficulties in directly measuring the influence of unlinked groups of examiners in different parallel versions of the exam. Consequently, little is known about how regional variations in examiners' judgements might challenge the equivalence of OSCEs (21) which could produce different outcomes for students in OSCE exams.

Two pre-requisites are necessary to determine equivalence within a distributed OSCE: firstly, common (or shared) OSCE content is needed, in order for examiners' judgements to be comparable, and secondly, a method is needed to compare examiners' scoring when they are distributed across different locations. In the UK, medical schools set their own OSCE exams, resulting in variation in content and format between Schools. Consequently, sharing OSCE content between schools, whilst necessary, will involve change from usual practice which could further influence examiner variability or produce unintended consequences.

Recently, Yeates et al (22–24) have iteratively developed a method to compare examiners' scoring within distributed OSCEs, called Video-based Examiner Score Comparison and Adjustment (VESCA). This produces linking of otherwise unlinked groups of examiners (termed "examiner-cohorts"(18) by 1/ videoing a small subset of students on each station of the OSCE; 2/ asking examiners from all examiner-cohorts to score the same station-specific comparator videos; and 3/ using the resulting score linkage to compare and equate for differences in examiner-cohorts. Their findings suggest that despite following accepted procedures for OSCE conduct, significant differences may persist between groups of examiners which could affect the pass/fail classification of a significant minority

For peer review only

of students. Follow-up work has enhanced the technique's feasibility (24), and shown that it is adequately robust to several potential confounding influences (25) and variations in implementation (26). Whilst these findings suggest that examiner-cohort effects are important and support the validity of VESCA for their measurement, VESCA has not yet been used across institutions, so both the likely magnitude of effects which may arise, and the practical implications of applying the method across institutions are unknown.

Finally, recent inquiry has focused on ensuring that trainees are competent across all relevant domains of performance(27), with a view to both providing diagnostic information to support their learning and enabling focused areas of deficit to be addressed rather than simply demonstrating a sufficient total score, as is often the case in OSCEs (28). This had led to scrutiny of the ability of OSCEs to prevent compensation between domains (29) and whether OSCEs could provide greater diagnosticity of students' areas of focal weakness. Whilst non-compensatory domain-based scoring has been trialled in other arenas (30), little is known about the psychometric properties of such domain scores or whether they can provide independent reliable scores for the constructs they represent. As the utility of VESCA would be greatly enhanced by providing domain level information which has been adjusted for the examiner-cohort effects, it is desirable to study the potential for these data to provide that information.

Collectively, it is anticipated that if these interventions are able to enhance the authenticity and equivalence of OSCEs whilst providing more diagnostic information on learners' performance, this will enhance OSCEs ability to support learning through their influence on students' preparation for OSCEs and their subsequent provision of more diagnostic feedback, whilst also ensuring greater confidence in the progression decisions which they inform. Consequently, understanding the interaction and use of these innovations is critical to determining their ability to benefit educational and healthcare practice.

For peer review only

Aims and Objectives

This project has a series of aims, objectives and research questions that set out to address the criticisms described above about OSCE examinations. These are:

Criticism 1: Lack of authenticity

- Objective 1: to increase perceived authenticity of an OSCE through use of integrated-task OSCE stations

Criticism 2: Examiner variability and challenges to equivalence

- Objective 2: to share integrated-task OSCE stations across different institutions and understand the implications which arise from the interaction of these stations with existing individual perceptions and institutional assessment practices.

Then, developing from that objective

- Objective 3: to use the VESCA methodology, within the context of a multi-centre integrated-task OSCE, to
    a. compare and equate for differences between examiner-cohorts in different institutions and
    b. understand the implications which arise from using VESCA across institutions.

Criticism 3: Limited diagnosticity of OSCEs across different domains of performance

- Objective 4: to determine whether different sub-domains of performance can be reliably distinguished from each other (rather than only providing an overall competence score) within a shared integrated-task OSCE.

For peer review only

Research Questions

Objectives 1 and 2 will be addressed jointly through research question 1

When integrated-task stations are used and shared within an OSCE, how, when, why and to what extent do examiners, students and simulated patients use and interact with them and how does this influence their perception of the authenticity of the OSCE scenarios?

Objective 3a will be addressed by the following research questions 2-5

2. How does the standard of examiners' judgements compare between examiner-cohorts?
3. How does the standard of examiners' judgements compare between institutions?
4. What are the relative magnitudes of inter versus intra institutional variation?
5. How much influence does adjusting for examiner-cohort effects have on students':
    a. Overall Scores
    b. Categorisation (fail / pass / excellence)
    c. Rank position

Objective 3b will be addressed through research question 6

When VESCA is used to compare and equate for differences between examiner-cohorts in different institutions within the context of a shared integrated-task OSCE, how, when, why and to what extent do examiners, students and simulated patients use and interact with VESCA?

Objective 4 will be addressed through research questions 7-8

7. How reliably can different domains of assessment be discriminated in unadjusted data?
8. Do students show differing patterns of performance across different domains of the assessment in unadjusted data?

For peer review only

Methods

Methodological Overview

The study will use a complex intervention design(31) to implement Video-based Examiner Score Comparison and Adjustment (VESCA) in the context of a multi-centre authentic-task OSCE. Research approaches will comprise psychometric analysis of assessment data(32) and Realist evaluation(33), collecting data through mixed methods. A schematic overview of the data collection and analysis is provided in Figure 1.

Population, Sampling and Recruitment

The study population will comprise participants of late years (penultimate and final year) undergraduate medical student clinical exams within the United Kingdom.

This population will be sampled by recruiting four medical schools to participate as centres in the study, with sampling from all relevant examiners, students, simulated patients. As no prior work has formally compared OSCE examination standards across UK medical schools, the study will aim to sample across different characteristics which might plausibly influence scoring: geographic divergence; Russell group and non-Russell group Universities; and new and more established medical schools.

Recruitment will be performed locally by each participating institution using both in-person and electronic advertisements. Each participating institute will have recruitment targets for students (n=24), examiners (n=12), and simulated patients (n=12). This sample size is pragmatic based on the resource implications for individual institutions of running a research OSCE. Whilst no formal method exists to power comparisons, or any agreed minimally important difference for differences between groups of OSCE examiners, subset analysis of data from Yeates et al 2021(24) suggests this

For peer review only

sample size is likely to provide a standard error in the region of 0.03 logits, enabling statistically significant detection of a difference between examiner cohorts of 5% of the assessment scale.

OSCE Design

The OSCE will comprise six tasks (stations). In each station, students will be directly observed for 13.5 minutes, with a further variable amount of preparation and rotation time of between 1.5 – 4 minutes per station, depending on each school's usual practice. Consequently, total testing time will range between 90-105 mins depending on different schools' practices.

Station content (simulated patient scenarios / instructions / stimulus materials / scoring rubrics) will be developed by the research team to reflect plausible simulated scenarios from Foundation year 1 doctors routine work and integrate multiple related processes which would be required for whole-task completion. For example, a station may describe a specific clinical scenario from the work of a new doctor and instruct candidates to perform a relevant clinical assessment. Candidates might then be expected to gather a clinical history, perform relevant focused physical examination, interpret provided investigation results, consult available guidelines and then describe their diagnosis and management to the patient. Tasks will be blueprinted against the UK General Medical Council's Clinical Skills Performance Assessment framework(34), to sample this framework's 3 domains: areas of clinical practice; clinical and professional capabilities; and areas of professional knowledge. The same stations will be used in all 4 study sites, whilst allowing minor adaptation for local contexts (for example by providing local antibiotic guidelines or dosage calculators).

Individual students will rotate around all 6 stations, and be observed by a different, single examiner in each station during a 90 minute "cycle" of the exam. Each site will host two parallel circuits of the OSCE (identical OSCE stations, run with different examiners). Twelve students will be examined in each parallel circuit (i.e. two cycles of 6 students), enabling 24 students to be tested at each site.

For peer review only

Examiners will be provided with station material (clinical scenario, simulated patient script, marking criteria) prior to the OSCE. Additionally, examiners will be provided with a web-link to a training video which will orientate them to the scoring format.

Examiners at all sites will score students' performances on the GeCoS rating system(35). This scoring system selects 5 appropriate performance domains for each station from a list of 20 when the station is designed (for example: history content, physical examination, clinical reasoning, building and maintaining the relationship, management content). Each domain is scored 1-4 (1=must improve; 2=borderline; 3=proficient; 4=very good). These scores are combined with a further 7-point global rating (1=incompetent; 7=excellent) to give a total score out of 27 for each station. Scoring will use tablet or paper-based marking based on available resources at each site.

The OSCE will be conducted first at the lead site (Keele) to enable video production for VESCA procedures; timing in other institutions will vary within an 8 month window to fit with local curricular demands. Local site teams will operationalise the station content based on the constraints of their local resources and equipment. Timing of stations will use local timing facilities but will adhere to standard timing intervals.

Intervention

VESCA will be employed using the methods developed by Yeates et al (22–24).

Video filming: Performances of all students in all 6 stations, from the first cycle, on a selected circuit, will be filmed at the lead site (Keele) using methods established by Yeates et al (23). Filming will use two unobtrusive wall-mounted closed-circuit TV cameras in every room (ReoLink 432, 1080 HD resolution). Camera position, angle and zoom will be selected to optimise capture of the performance. Sound will be recorded using a stereo condensing boundary microphones (Audio-Technica Pro 44). The first three videos from each station which are technically adequate

For peer review only

(unobstructed pictures with adequate sound) will be selected and processed for further use, resulting in three comparison videos for each of the six stations in the OSCE.

Video scoring: All examiners will be asked to score the three selected videos selected for the station they examine. All examiners who examine a given station will score the same videos. To facilitate this, videos will be securely shared across institutions, using the secure on-line video scoring approach developed by Yeates et al(24). This will include the following elements: on-line consent; station-specific examiner information; sequential presentation of the 3 comparison videos for the station. Examiners will have to score each video and provide brief feedback before progressing to the next. As per Yeates et al (23), examiners will have 4 weeks after the OSCE to complete video scoring.

Data Collection

Student scores (live and video performances) from each site will be collated and labelled with unique identifiers indicating 1/ student, 2/ site, 3/ circuit, 4/ station, 5/examiner, 6/ examiner-cohort and 7/ video or live performance. These data will be used to address all psychometric research questions.

To address research questions 1 & 6, researchers will develop an initial programme theory (IPT)(36) to orientate and focus subsequent data collection and analysis. To develop the initial programme theory, researchers will consider prior research on VESCA, published experiences of international multi-institutional OSCE collaborations, formal theories which concern institutional adoption of innovations, and the views of a range of experience assessment professionals.

Data will be collected iteratively, interspersed by analysis(37), through a mixture of observation, individual interviews (38) and (where feasible) focus groups, supplemented by available process data. This, along with score data, will be triangulated across modalities to support validity.

Interviews will sample individuals from all relevant stakeholder groups at each site, focused on individuals who have participated in the research OSCE. Whilst sampling requirements will be data

For peer review only - http://bmjopen.bmj.com/site/about/guidelines.xhtml

driven, indicative numbers of each group from each site are students (n=4), examiners (n=4), simulated patients(n=3), and OSCE administrators(n=1-2). All individuals participating in the OSCE will be invited to be interviewed. If offers of participation exceed sampling needs, then participants will be selected to maximise sample representativeness. Recruitment will be performed by email. Participation will be voluntary. Participants will receive study information and asked to record their consent through an on-line consent form. Interviews will be conducted by members of the research team (PI, or research assistants), and are expected to last 45-60 mins. Interviews will be conducted in-person in a private place or via Microsoft Teams. Interviews will be audio recorded and professionally transcribed. Interviews will be guided by a topic guide which will draw from the IPT and evolving theory and will be illustrated by practice-based examples where needed. The interview approach will be adapted to glean, refine and then consolidate emerging theory (39).

Two researchers will observe the “on-the-day” conduct of the OSCE in each participating medical school, using Realist ethnographic observation methods (40). As far as feasible this will include: preparation for the OSCE, including station layout, equipment set-up, timing and scoring methods; conduct of the OSCE, including student flow around the circuits and observation of students examiners and simulated patients behaviour and interactions during and between station performances; students and examiners interaction with filming; and participants’ responses to both the OSCE and VESCA in breaks or after the OSCE is complete. Researchers’ observations will be recorded through field notes which may be supplemented by examples of items or materials from the OSCE, diagrams or photographs.

Process data will be collected by researchers from each school depending on availability and may include participant recruitment data, score data, website metrics from examiner training materials and metrics related to video scoring by examiners.

For peer review only

Patient and Public Involvement

Patients and members of the public have been involved throughout the VESCA programme of research which has led to this study. This has included establishing the priority of the research, reviewing plain English summaries, contributing to the design of the research, reviewing progress contributing to elements of the analysis and interpreting findings and discussing future directions. Members of the public are expected to contribute to dissemination activities.

Analysis

For peer review only

Realist Analyses (used for data relating to research questions 1 & 6)

Similar analysis methods will be used for both questions. Audio recordings of interviews and focus groups will be professionally transcribed. Observation field notes, where available, will be incorporated into the dataset as will summaries of score data, participation rates and engagement metrics from on-line video scoring by examiners and video access metrics from the on-line feedback portal for students. Analysis will use the stages described by Papoutsi et al (41). This begins by reading or considering each piece of data line-by-line to judge its relevance to the initial programme theory. Next, where needed, decisions will be made about the trustworthiness of relevant data. Next, researchers will allocate initial conceptual labels. Conceptual labels will be derived both deductively from the initial programme theory and inductively based on researchers' interpretation of emergent issues. Researchers will then consider whether each labelled concept can be interpreted to represent a context (C), a mechanism (M) or an outcome (O) and will look for data which provides information on the relatedness of Cs, Ms, and Os, so that they may be developed into Context-Mechanism-Outcome-Configurations (CMOC). Drawing on relevant data, researchers will then interpret how each CMOC relates to the programme theory and iteratively revise the programme theory as more and more CMOCs are developed. Interpretation will use the analytic processes of juxtaposition, reconciliation, adjudication and consolidation to explore discrepancies

and resolve differences. Interpretation will also use retro-duction, combining both induction based on emergence from the data and deduction from the initial programme theory in order to unearth mechanistic relations within CMOCs and the Programme theory(42,43). Analysis will proceed iteratively, interspersed with new data collection until a coherent and plausible programme theory is reached.

Psychometric analyses (used for RQs 2-5, 7,8)

Research questions 2-5 will be addressed using Many Facet Rasch Modelling (MFRM), conducted using FACETs by Winsteps (44). The dependent variable for analyses will be denoted "total score" and will be calculated for each student on each station by combining the scores for each domain. Categorical independent variables will be available for each station score, describing the student (unique ID number); station (station number); examiner (examiner ID); examiner-cohort (ex-cohort ID); and site (institution ID). These data will be analyses using a four facet Rasch model, with facets of: 1/ student, 2/ station, 3/ examiner-cohort and 4/ site.

To ensure data are adequate for MFRM analysis, research will assess the dimensionality, ordinality and model-fit of data. Dimensionality will be assessed using principle components analysis (PCA) of model residuals with random parallel analysis using R studio for R(45). Ordinality of the scale will be determined by examining the Rasch-Andrich thresholds supplied in FACETs output data(FACETS v3.82.3 Winsteps, Western Australia). Fit parameters supplied by FACETs will be examined to determine data to model fit, using the criteria advocated by Linacre (46). If data are inadequate for MFRM analysis, then the analysis plan will be adapted to use an appropriate alternative method such as linear mixed modelling.

To explore the potential that differences in institutional implementation of the OSCE might confound the measurement of examiner-cohort effects between institutions, we will additionally compare examiner cohort effects on the subset of score data arising from examiners' video scoring. This will offer a controlled comparison (as all examiner cohorts will score the same video

For peer review only

performances). Analysis will use generalised linear modelling (GLiM), including only data from examiners scoring of videos. The dependent variable will be total score, with factors of: station, examiner-cohort, and school will be included in the model. Results from this analysis will be presented alongside the main analysis, to enable the likelihood of bias in the MFRM to be judged as part of overall evaluation of the complex intervention.

To address RQ2, observed (Raw score) average scores and "Fair-Average" scores(47) for examiner-cohorts will be compared, and the difference between observed (Raw score) average and Fair average will be calculated for each examiner-cohort and compared. Observed differences will be transformed into multiples of the standard error to calculate statistical significance.

To address RQ3 observed (Raw score) average scores and "Fair-Average" scores(47) for each site (institution) will be compared and the difference between their observed (Raw score) average and Fair average will be calculated for each site and compared

To address RQ4, the difference between examiner-cohorts within each institution (i.e. site) will be calculated and compared with the differences between the values for different institutions

To address RQ5a, the difference between the raw observed average score and the fair average score will be calculated for each participating student. These will be converted to mean absolute differences (MAD) to remove the direction of score adjustment. Descriptive statistics will be calculated for both the raw score adjustments and MAD adjustments. Similar to prior research (22,24), the effect size of each MAD score adjustment will calculated using Cohen's d (48), using the standard deviation of students' average observed scores as the denominator. The mean Cohen's d and the proportion of students' whose adjustment exceeds d=0.5 will be reported.

To address RQ5b&c, category boundaries will be developed using the borderline regression method(49) for each station and pooled to give an average cut score for the test. Two separate values will be interpolated from the x-axis: one to represent a fail/pass boundary and one to

For peer review only

represent a pass/excellent boundary. Each students' categorisation for the OSCE relative to these boundaries will be determined based on their observed raw average score and their fair average score and the proportion changing categories (number increasing a grade; number reducing a grade) will be calculated for both thresholds. Students rank position in the OSCE (regardless of institutional rankings) will be calculated based on observed raw average scores and fair average scores and the difference between each student's rank position from each score calculated. This will be expressed as both raw change in rank (positive or negative sign) and MAD change in rank which will be summarised through descriptive statistics.

Research questions 7&8 represent exploratory forms of analysis. These analyses will use the scores in individual scores domains within each station as dependent variables. Domains will be grouped based on content into dimensions which represent communication skills, knowledge and reasoning, investigation and management and procedural skills. Exploratory Factor Analysis will be used to determine the level of support for these dimensions, and Cronbach's alpha will be used to estimate the reliability of scores within each dimension. Student-level dimension scores will be examined to produce descriptive statistics describing dimension level scores and to determine the proportion of students who show greater than 0.5 standard deviation score difference between difference dimensions. Further exploratory analyses will determine whether categorical differences exist for some students across domains (i.e. greater frequency of borderline categories in 1 domain).

For peer review only

Anticipated Outcomes

Realist evaluations will produce mature programme theories which describe how different contexts elicit different mechanisms to produce varied outcomes for different stakeholders when a/ an integrated authentic task OSCE is shared between medical schools and b/. VESCA is implement across multiple medical schools. This will be used to produce guidance on successful implementation

of both interventions. Realist Evaluations will be reported using the standards of the RAMESES II reporting standards(38).

Psychometric analyses for RQs 2-5 will describe the extent of overall score variability which arose between examiner-cohorts and institutions in the standard of examiners' judgements, and the impact of adjustment for these on students' scores, categorisation and rank.

Psychometric analyses for RQs 7&8 will describe the dimensionality of domain-score data and varied patterns of strength and weakness in students' performances, with comparison in patterns across schools.

Ethics and Dissemination

This study will recruit volunteer students, examiners and SPs. Recruitment will invite the entirety of relevant students and examiner populations, subject to any local exclusions (for example adequate academic progress). Simulated patients will participate as per their usual professional working arrangements. Participants will retain the right to withdraw up until their data are anonymised after which point withdrawal will not be possible. Researchers will collect personal data to manage recruitment and to link scores from the OSCE, on-line usage and engagement data for video access or scoring and interview and focus group data. These data will be stored securely and treated as confidential. Access will be limited to those members of the research team who require access for the analyses specified within the research. Participants will be asked to indicate whether they permit their data to be used in future research or to be contacted about future research. There are few anticipated risks to participants: if videos, score or interview data pertaining to them were disseminated inadvertently then that could cause embarrassment or distress. This risk is mitigated through the confidentiality and data security measures which will be employed. Students may benefit from taking part in the research through the experience of novel OSCE assessment tasks or

For peer review only

availability of video feedback. Examiners may benefit from practice at examining. Ethical approval for the study has been granted by Keele University Research Ethics committee (Ref: MH-210209)

Study reporting will describe the blue printing and station development process; scoring format; an overview of station content and test reliability.

Findings of the research will be disseminated through academic publications, conference presentations and workshops and through engagement meetings with educational institutions who may adopt or implement VESCA or Video-based feedback.

Outputs

Good practice guidelines for the use of VESCA to enhance OSCE examiner standardisation in distributed exams & for sharing integrated task OSCEs across institutions. Intended audiences: institutions, assessment leads, examiners. Engagement work through the Association for the Study of Medical Education Psychometrics Specialist Interest Group (ASME psychoSIG) to promote this to policy makers.

Explanatory video, describing the purpose, use and benefits of VESCA for a lay audience. Intended audience, students, examiners, members of the public.

Publications

The research is expected to produce academic publications describing the following findings:

1. Paper 1: primary psychometric analyses, comparing the influence of examiner-cohorts and institutions on students' scores, categorisation and rank
2. Paper 2: secondary psychometric analyses, determining the extent of additional diagnostic information available in domain score data.
3. Paper 3: Realist evaluation, a programme theory of the implications of using integrated-task OSCE stations to increase authenticity in OSCE and using VESCA within a shared OSCE.

For peer review only

Anticipated Timeframe

Developing collaborations: complete by end May 2021.

Finalising protocol: June 2021

Ethics application: July-Sept 2021

OSCE station development: September - October 2021

Scheduling and recruitment of OSCEs: October 2021 – March 2022

Site 1 OSCE: December – March 2022

Sites 2-4 OSCE: January – July 2022

Examiner video scoring: 4-week interval after each OSCE

Interviews / focus groups / observations: December – August 2022

Psychometric analyses: July – November 2022

Realist analysis February - November 2022

Dissemination: December 2022 - February 2023.

Contributorship

The study design was developed by PY, RK, NC, GM, KC, VO, AC, RG, RV, CWC, RKM and RF. PY wrote the original draft. GW provided expertise in Realist Evaluation methodology. PY, AM and NC are collecting data supported by KC, RV, CWC, RG, RV. PY and AM will analyse the data. All authors critiqued and provided edits to the manuscript for intellectual content.

For peer review only

Funding Statement

The study is funded through a National Institute for Health Research (NIHR) Clinician Scientist award held by the Principal Investigator, Peter Yeates. The study constitutes independent research and does not represent the views of the NIHR, the NHS or the department of health and social care.

Competing of Interests

There are no competing interests for any author.

References

1. Valentine N, Durning S, Shanahan EM, Schuwirth L. Fairness in human judgement in assessment: a hermeneutic literature review and conceptual framework. Adv Heal Sci Educ. 2020;26(2):713–38.

2. Eva KW. Cognitive Influences on Complex Performance Assessment: Lessons from the Interplay between Medicine and Psychology. J Appl Res Mem Cogn. 2018;7(2):177–88.

3. Kogan JR, Conforti LN, Iobst WF, Holmboe ES. Reconceptualizing variable rater assessments as both an educational and clinical care problem. Acad Med. 2014 May;89(5):721–7.

4. Newble D. Techniques for measuring clinical competence: objective structured clinical examinations. Med Educ. 2004;38:199–203.

5. Boursicot K, Kemp S, Wilkinson T, Findyartini A, Canning C, Cilliers F, et al. Performance assessment: Consensus statement and recommendations from the 2020 Ottawa Conference. Med Teach. 2021 Jan 2;43(1):58–67.

For peer review only

6. Harden RM, Stevenson M, Downie WW, Wilson GM. Medical Education Assessment of Clinical Competence using Objective Structured Examination. Br Med J. 1975;1(February):447–51.

7. Norcini J, Anderson MB, Bollela V, Burch V, Costa MJ, Duvivier R, et al. 2018 Consensus framework for good assessment. Med Teach. 2018;0(0):1–8.

8. Wass V, Vleuten C Van Der, Shatzer J, Jones R. Medical education quartet Assessment of clinical competence. Lancet. 2001;357:945–9.

9. Huffman BM, Hafferty FW, Bhagra A, Leasure EL, Santivasi WL, Sawatsky AP. Resident impression management within feedback conversations: A qualitative study. Med Educ. 2021;55(2):266–74.

10. Murto SH, Shaw T, Touchie C, Pugh D, Cowley L, Wood TJ. Are raters influenced by prior information about a learner ? A review of assimilation and contrast effects in assessment. Adv Heal Sci Educ. 2021;(0123456789).

11. Johnston JL, Kearney GP, Gormley GJ, Reid H. Into the uncanny valley: Simulation versus simulacrum? Med Educ. 2020;54(10):903–7.

12. Gormley GJ, Hodges BD, McNaughton N, Johnston JL. The show must go on? Patients, props and pedagogy in the theatre of the OSCE. Med Educ. 2016;50(12):1237–40.

13. Downing SM. Validity: on meaningful interpretation of assessment data. Med Educ. 2003 Sep;37(9):830–7.

14. Ruesseler M, Weinlich M, Byhahn C, Müller MP, Jünger J, Marzi I, et al. Increased authenticity in practical assessment using emergency case OSCE stations. Adv Heal Sci Educ. 2010;15(1):81–95.

15. Schoenmakers B, Wens J. The objective structured clinical examination revisited for

For peer review only

postgraduate trainees in general practice. Int J Med Educ. 2014;5:45–50.

16. Gormley G, Sterling M, Menary A, McKeown G. Keeping it real! Enhancing realism in standardised patient OSCE stations. Clin Teach. 2012;9(6):382–6.

17. Gingerich A. The Reliability of Rater Variability. J Grad Med Educ. 2020;12(2):159–61.

18. Yeates P, Sebok-Syer SS. Hawks, Doves and Rasch decisions: Understanding the influence of different cycles of an OSCE on students' scores using Many Facet Rasch Modeling. Med Teach. 2017;39(1):92–9.

19. Sebok SS, Roy M, Klinger D a, De Champlain AF. Examiners and content and site: Oh My! A national organization's investigation of score variation in large-scale performance assessments. Adv Health Sci Educ Theory Pract. 2015 Aug 28;20(3):581–94.

20. Floreck LM, De Champlain AF. Assessing Sources of Score Variability in a Multi-Site Medical Performance Assessment: An Application of Hierarchical Linear Modeling. Acad Med. 2001;76(10):S93–5.

21. Norcini J, Anderson B, Bollela V, Burch V, Costa MJ, Duvivier R, et al. Criteria for good assessment: consensus statement and recommendations from the Ottawa 2010 Conference. Med Teach. 2011 Jan;33(3):206–14.

22. Yeates P, Cope N, Hawarden A, Bradshaw H, McCray G, Homer M. Developing a video-based method to compare and adjust examiner effects in fully nested OSCEs. Med Educ. 2019 Mar;53(3):250–63.

23. Yeates P, Moult A, Lefroy J, Walsh-House J, Clews L, McKinley R, et al. Understanding and developing procedures for video-based assessment in medical education. Med Teach. 2020 Nov 1;42(11):1250–60.

24. Yeates P, Moult A, Cope N, McCray G, Xilas E, Lovelock T, et al. Measuring the Effect of

For peer review only

Examiner Variability in a Multiple-Circuit Objective Structured Clinical Examination (OSCE). Acad Med. 2021 Mar 2;96(8):1189–96.

25. Yeates P, Moult A, Cope N, McCray G, Fuller R, McKinley R. Determining influence, interaction and causality of contrast and sequence effects in objective structured clinical exams. Med Educ. 2022;56(3):292–302.

26. Yeates P, McCray G, Moult A, Cope N, Fuller R, McKinley R. Determining the influence of different linking patterns on the stability of students' score adjustments produced using Video-based Examiner Score Comparison and Adjustment (VESCA). BMC Med Educ. 2022;22(1):1–9.

27. Frank JR, Snell LS, Cate O Ten, Holmboe ES, Carraccio C, Swing SR, et al. Competency-based medical education: theory to practice. Med Teach. 2010 Aug 27;32(8):638–45.

28. McKinley DW, Norcini JJ. How to set standards on performance-based examinations: AMEE Guide No. 85. Med Teach. 2014;36(2):97–110.

29. Homer M, Russell J. Conjunctive standards in OSCEs: The why and the how of number of stations passed criteria. Med Teach. 2021;43(4):448–55.

30. Pearce J, Reid K, Chiavaroli N, Hyam D. Incorporating aspects of programmatic assessment into examinations: Aggregating rich information to inform decision-making. Med Teach. 2021 Feb 8;0(0):1–8.

31. Craig P, Dieppe P, Macintyre S, Michie S, Nazareth I, Petticrew M. Developing and evaluating complex interventions : new guidance. 2008.

32. Bond T, Fox C. Applying the Rasch Model Fundamental Measurement in the Human Sciences. 2nd Editio. New York & London: Routledge; 2012.

33. Pawson R, Tilley N. Realistic Evaluation. 1st ed. London: Sage Publications Ltd; 1997.

For peer review only

34. General Medical Council. MLA content map.

35. Lefroy J, Gay SP, Gibson S, Williams S, McKinley RK. Development and face validation of an instrument to assess and improve clinical consultation skills. Int J Clin Ski. 2011;5(2):115–125.

36. Pawson R, Manzano-Santaella A. A realist diagnostic workshop. Evaluation. 2012;18(2):176–91.

37. Marchal B, van Belle S, van Olmen J, Hoerée T, Kegels G. Is realist evaluation keeping its promise? A review of published empirical studies in the field of health systems research. Evaluation. 2012;18(2):192–212.

38. Wong G, Westhorp G, Manzano A, Greenhalgh J, Jagosh J, Greenhalgh T. RAMESES II reporting standards for realist evaluations. BMC Med. 2016;14(1):1–18.

39. Manzano A. The craft of interviewing in realist evaluation. Evaluation. 2016;22(3):342–60.

40. Hammersley M. Ethnography and Realism. In: Huberman AM, Miles MB, editors. The Qualitative Researcher's Companion. 2455 Teller Road, Thousand Oaks California 91320 United States of America: SAGE Publications, Inc.; 2002. p. 65–80.

41. Papoutsi C, Mattick K, Pearson M, Brennan N, Briscoe S, Wong G. Interventions to improve antimicrobial prescribing of doctors in training (IMPACT): a realist review. Heal Serv Deliv Res. 2018;6(10):1–136.

42. Astbury B, Leeuw FL. Unpacking Black Boxes: Mechanisms and Theory Building in Evaluation. Am J Eval. 2010;31(3):363–81.

43. The RAMESES II Project. Retroduction in realist evaluation. Nihr. 2017;(p 207):1–3.

44. Linacre JM. Many-Facet Rasch Measurement. 2nd Edicat. Chicago: MESA Press; 1994.

45. Team Rs. RStudio: Integrated Development for R. Boston, MA: RStudio, Inc.; 2015.

For peer review only

46. Linacre JM. What do Infit and Outfit, Mean-square and Standardized mean? [Internet]. Rasch.Org website. 2002 [cited 2018 Jun 12]. p. 16:2, p878. Available from: https://www.rasch.org/rmt/rmt162f.htm

47. Linacre JM. A User's guide to FACETS Rasch-Model Computer Programs. 2005.

48. Cohen J. Statistical Power Analysis for the Social Sciences. 2nd ed. Lawrence Erlbaum Associates; 1988.

49. Downing SM, Tekian A, Yudkowsky R. Procedures for Establishing Defensible Absolute Passing Scores on Performance Examinations in Health Professions Education Procedures for Establishing Defensible Absolute Passing Scores on Performan. Teach Learn Med. 2006;18(1):50–7.

Figure 1: Schematic of the data collection and analysis processes

For peer review only

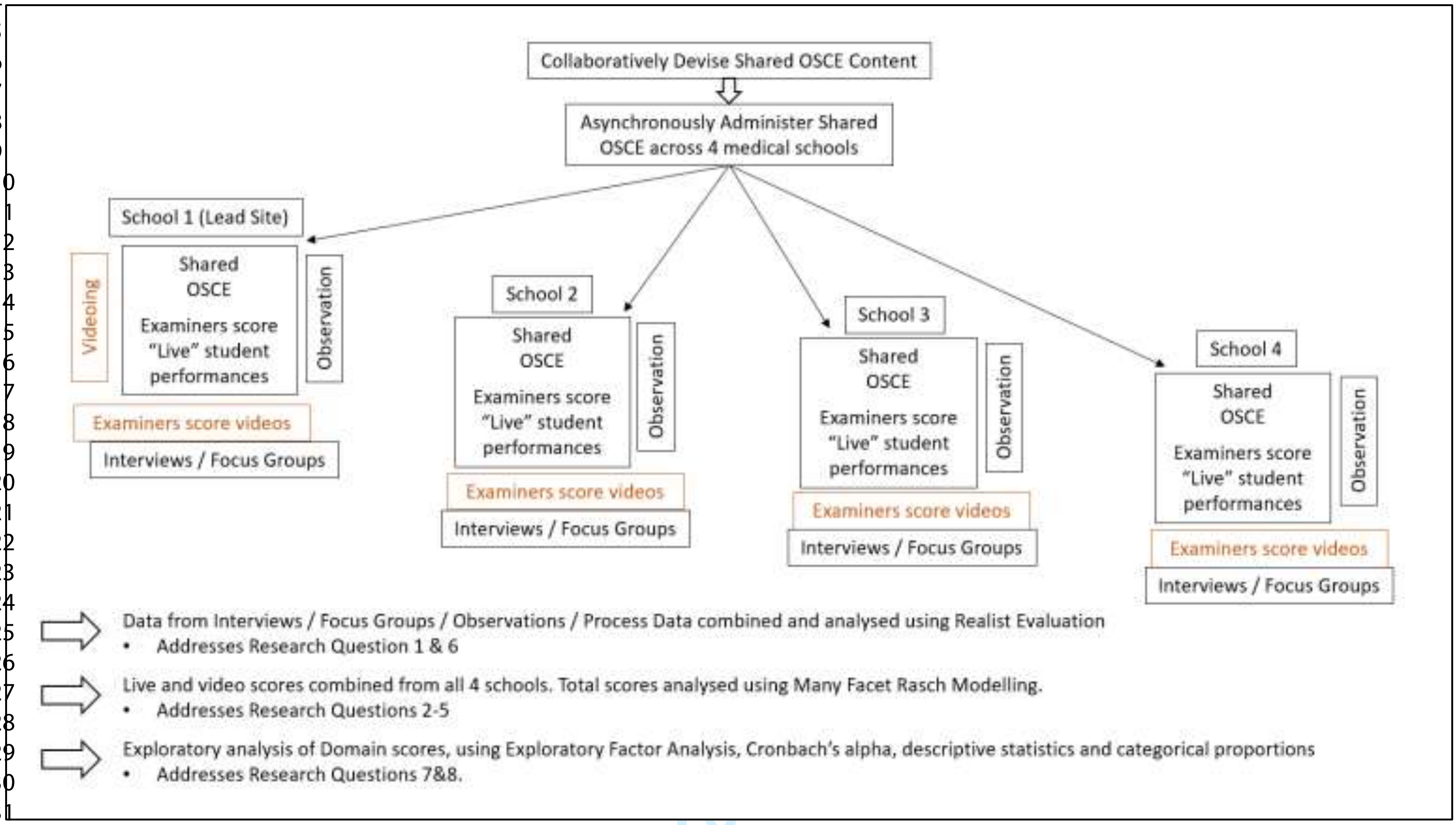

For peer review only

CONSORT 2010 checklist of information to include when reporting a randomised trial*

Section/Topic	Item No	Checklist item	Reported on page No
**Title and abstract**			
	1a	Identification as a randomised trial in the title	n/a
	1b	Structured summary of trial design, methods, results, and conclusions (for specific guidance see CONSORT for abstracts)	1-2
**Introduction**			
Background and objectives	2a	Scientific background and explanation of rationale	3-5
	2b	Specific objectives or hypotheses	5-7
**Methods**			
Trial design	3a	Description of trial design (such as parallel, factorial) including allocation ratio	7-10
	3b	Important changes to methods after trial commencement (such as eligibility criteria), with reasons	n/a
Participants	4a	Eligibility criteria for participants	10
	4b	Settings and locations where the data were collected	10
Interventions	5	The interventions for each group with sufficient details to allow replication, including how and when they were actually administered	10-11
Outcomes	6a	Completely defined pre-specified primary and secondary outcome measures, including how and when they were assessed	n/a
	6b	Any changes to trial outcomes after the trial commenced, with reasons	n/a
Sample size	7a	How sample size was determined	10-11
	7b	When applicable, explanation of any interim analyses and stopping guidelines	n/a
Randomisation:			
Sequence generation	8a	Method used to generate the random allocation sequence	n/a
	8b	Type of randomisation; details of any restriction (such as blocking and block size)	n/a
Allocation concealment mechanism	9	Mechanism used to implement the random allocation sequence (such as sequentially numbered containers), describing any steps taken to conceal the sequence until interventions were assigned	n/a
Implementation	10	Who generated the random allocation sequence, who enrolled participants, and who assigned participants to interventions	n/a
Blinding	11a	If done, who was blinded after assignment to interventions (for example, participants, care providers, those	n/a

For peer review only

For peer review only - http://bmjopen.bmj.com/site/about/guidelines.xhtml

		assessing outcomes) and how	
	11b	If relevant, description of the similarity of interventions	4
Statistical methods	12a	Statistical methods used to compare groups for primary and secondary outcomes	12-14
	12b	Methods for additional analyses, such as subgroup analyses and adjusted analyses	11-12
**Results**			
Participant flow (a diagram is strongly recommended)	13a	For each group, the numbers of participants who were randomly assigned, received intended treatment, and were analysed for the primary outcome	n/a
	13b	For each group, losses and exclusions after randomisation, together with reasons	n/a
Recruitment	14a	Dates defining the periods of recruitment and follow-up	17
	14b	Why the trial ended or was stopped	n/a
Baseline data	15	A table showing baseline demographic and clinical characteristics for each group	n/a
Numbers analysed	16	For each group, number of participants (denominator) included in each analysis and whether the analysis was by original assigned groups	n/a
Outcomes and estimation	17a	For each primary and secondary outcome, results for each group, and the estimated effect size and its precision (such as 95% confidence interval)	n/a
	17b	For binary outcomes, presentation of both absolute and relative effect sizes is recommended	n/a
Ancillary analyses	18	Results of any other analyses performed, including subgroup analyses and adjusted analyses, distinguishing pre-specified from exploratory	15-16
Harms	19	All important harms or unintended effects in each group (for specific guidance see CONSORT for harms)	16
**Discussion**			
Limitations	20	Trial limitations, addressing sources of potential bias, imprecision, and, if relevant, multiplicity of analyses	n/a
Generalisability	21	Generalisability (external validity, applicability) of the trial findings	15
Interpretation	22	Interpretation consistent with results, balancing benefits and harms, and considering other relevant evidence	11-14
**Other information**			
Registration	23	Registration number and name of trial registry	n/a
Protocol	24	Where the full trial protocol can be accessed, if available	n/a
Funding	25	Sources of funding and other support (such as supply of drugs), role of funders	22

*We strongly recommend reading this statement in conjunction with the CONSORT 2010 Explanation and Elaboration for important clarifications on all the items. If relevant, we also recommend reading CONSORT extensions for cluster randomised trials, non-inferiority and equivalence trials, non-pharmacological treatments, herbal interventions, and pragmatic trials. Additional extensions are forthcoming: for those and for up to date references relevant to this checklist, see www.consort-statement.org.11-114

For peer review only - http://bmjopen.bmj.com/site/about/guidelines.xhtml
